# Strathclyde minor groove binders (S-MGBs) with activity against *Acanthamoeba castellanii*

**DOI:** 10.1093/jac/dkae221

**Published:** 2024-07-09

**Authors:** Leah M C Mcgee, Alemao G Carpinteyro Sanchez, Marina Perieteanu, Kaveh Eskandari, Yan Bian, Logan Mackie, Louise Young, Rebecca Beveridge, Colin J Suckling, Craig W Roberts, Fraser J Scott

**Affiliations:** Department of Pure and Applied Chemistry, University of Strathclyde, Glasgow, UK; Strathclyde Institute of Pharmacy and Biomedical Sciences, University of Strathclyde, Glasgow, UK; Department of Pure and Applied Chemistry, University of Strathclyde, Glasgow, UK; Department of Pure and Applied Chemistry, University of Strathclyde, Glasgow, UK; Department of Pure and Applied Chemistry, University of Strathclyde, Glasgow, UK; Strathclyde Institute of Pharmacy and Biomedical Sciences, University of Strathclyde, Glasgow, UK; Strathclyde Institute of Pharmacy and Biomedical Sciences, University of Strathclyde, Glasgow, UK; Department of Pure and Applied Chemistry, University of Strathclyde, Glasgow, UK; Department of Pure and Applied Chemistry, University of Strathclyde, Glasgow, UK; Strathclyde Institute of Pharmacy and Biomedical Sciences, University of Strathclyde, Glasgow, UK; Department of Pure and Applied Chemistry, University of Strathclyde, Glasgow, UK

## Abstract

**Background:**

*Acanthamoeba* spp. is the causative agent of *Acanthamoeba* keratitis and granulomatous amoebic encephalitis. Strathclyde minor groove binders (S-MGBs) are a promising new class of anti-infective agent that have been shown to be effective against many infectious organisms.

**Objectives:**

To synthesize and evaluate the anti-*Acanthamoeba* activity of a panel of S-MGBs, and therefore determine the potential of this class for further development.

**Methods:**

A panel of 12 S-MGBs was synthesized and anti-*Acanthamoeba* activity was determined using an alamarBlue^™^-based trophocidal assay against *Acanthamoeba castellanii*. Cross-screening against *Trypanosoma brucei brucei*, *Staphylococcus aureus* and *Escherichia coli* was used to investigate selective potency. Cytotoxicity against HEK293 cells allowed for selective toxicity to be measured. DNA binding studies were carried out using native mass spectrometry and DNA thermal shift assays.

**Results and discussion:**

S-MGB-241 has an IC_50_ of 6.6 µM against *A. castellanii*, comparable to the clinically used miltefosine (5.6 µM) and negligible activity against the other organisms. It was also found to have an IC_50_ > 100 µM against HEK293 cells, demonstrating low cytotoxicity. S-MGB-241 binds to DNA as a dimer, albeit weakly compared to other S-MGBs previously studied. This was confirmed by DNA thermal shift assay with a Δ*T*_m_ = 1 ± 0.1°C.

**Conclusions:**

Together, these data provide confidence that S-MGBs can be further optimized to generate new, potent treatments for *Acanthameoba* spp. infections. In particular, S-MGB-241, has been identified as a ‘hit’ compound that is selectively active against *A. castellanii*, providing a starting point from which to begin optimization of DNA binding and potency.

## Introduction

*Acanthamoeba* spp. are among the most prevalent free-living amoebae, occurring in many natural environments, but also thrive in man-made habitats.^1^ This amoeba is amphizoic being able to feed on bacteria, algae and yeasts, but importantly is also a facultative and opportunistic parasite of humans. It can live under extreme conditions concerning pH, level of oxygen, salinity and temperature. The life cycle of *Acanthamoeba* comprises two stages: an infective trophozoite stage that feeds and multiplies, and a dormant resistant cyst stage, which allows the amoeba not only to withstand harsh conditions, but also resist disinfection in the environment and drug therapy when parasitic.^2^

As a facultative parasite, *Acanthamoeba* spp. is the causative agent of a painful sight-threatening infection known as *Acanthamoeba* keratitis (AK) occurring mostly in contact lens wearers.^3^ Granulomatous amoebic encephalitis (GAE), is a result of opportunistic infection in immunodeficient patients and is almost always fatal. It is sometimes associated with disseminated infection that typically shows as skin infection or inflammation of the lungs and sinuses.^4^ Despite low incidence, patients infected with AK experience severe morbidity, which can be compounded by late diagnosis. Successful treatment of GAE is rare, but options used include treatment of underlying immunodeficiency, or a combination of chemotherapy, surgery and cryotherapy.^5,6^ Cases of AK are increasing due to a lack of awareness in the clinic, the deficiency of rapid diagnostic tools and increased numbers of people wearing contact lenses.^7^ Current therapies for AK are arduous as they require the repeated topical application of drugs to the cornea. In the UK, a combination of biguanide (PHMB or Chlorhexidine) and a diamidine (propamidine or hexamidine) are administered hourly day and night for the first 48 hours; this is continued hourly during daytime only for 2 weeks and then every 2 hours for at least 3–4 weeks.^8^ More recently, for disease that is unresponsive, miltefosine has been administered orally as an adjuvant systemic therapy.^9^ Even this does not reliably result in medical cure and can leave the cornea sufficiently damaged to require a keratoplasty. Thus, there is an urgent need to create new improved compounds to treat these diseases and avoid the worst patient outcomes.

**Figure 1.**
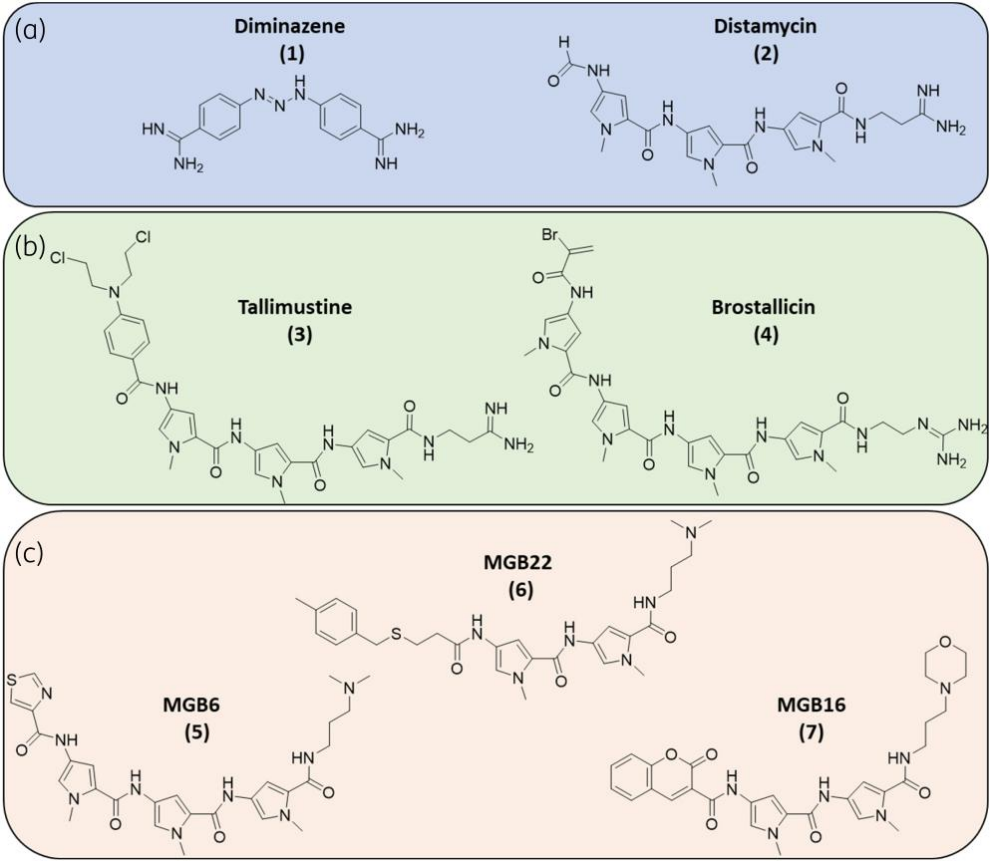
Structures of minor groove binders. (a) MGBs which interact with DNA via non-covalent interactions diminazene, **1**, and distamycin, **2**. (b) alkylating MGBs tallimustine, **3,** and brostallicin, **4**. (c) MGBs with anti-acanthamoeba properties, MGB6, **5**, MGB16, **6** and MGB22, **7**, as reported by Alniss *et al.*^10^ This figure appears in colour in the online version of *JAC* and in black and white in the print version of *JAC*.

Minor groove binders (MGBs) are small molecules that bind to the minor groove of DNA and consequently interrupt various DNA-centric processes. There are several structural classes of MGB, including the anti-parasitic diamidines, such as diminazene, **1**, a treatment for animal African trypanosomiasis, and also derivatives of the natural product distamycin, **2**. These compounds typically interact with the minor groove of DNA through non-covalent interactions and thus are able to bind reversibly without damaging DNA. However, several anti-cancer MGBs do contain an alkylating moiety, such as tallimustine, **3**, or brostallicin, **4**, which imparts additional safety concerns that are not relevant for non-alkylating MGBs (Figure 1).

The Strathclyde MGB (S-MGB) drug discovery platform is based on the template of distamycin, **2**. Over many iterations, the structure has been optimized to yield compounds with various anti-infective properties, including anti-parasitic, anti-viral, antibacterial, anti-mycobacterial and anti-fungal.^10–13^ Like distamycin, S-MGBs are able to bind to AT-rich sequences of DNA, enabling these compounds to target multiple loci on a pathogen’s DNA.^14^ Thus, S-MGBs fully embrace the multitargeted anti-infective drug concept, advantageous in this antimicrobial resistance era due to the reliance to target-based resistance it brings.^15^ Indeed, several studies have confirmed that S-MGBs are resilient to the generation of resistance in laboratory experiments.^16^ Our continued interest in expanding the clinical potential of S-MGBs lead us to investigate their effects against the amoeba *A. castellanii*.

In parallel with our present studies, Alniss *et al.* have also investigated and disclosed distamycin derived MGBs with anti-*Acanthamoeba* properties (Figure 1c).^17^ 12 structurally diverse MGBs were synthesized that explored the significance of features such as head group lipophilicity, tail group basicity and heterocycle substitutions. The MGBs’ amoebicidal activity and effects on encystation and excystation were studied. Amoeba-mediated host-cell death was also investigated through cytopathogenicity assays. Ultimately, their MGB6, **5**, MGB16, **6**, and MGB22, **7**, were found to be most potent in their assays cascade, noting that MGB6, **5,** had the most potent IC_50_ of 58 µM in the amoebicidal assay.

In our study, 12 S-MGBs, including zwitterionic structures, were synthesized and evaluated for activity against several pathogens, including *Acanthamoeba castellanii*, *Trypanosoma brucei*, *Staphylococcus aureus* and *Escherichia coli.* Zwitterionic structures were of interest as they have not been explored in our extensive studies on S-MGB structure activity relationships. Additionally, the compounds in this set adopt our recent truncated S-MGB strategy, shortening the overall length of the molecules, which has previously shown to result in selectivity towards the parasitic organisms, including *Trypanosoma* spp. and *Leishmania* spp., over other bacterial pathogens, *S. aureus* and *E. coil*.^18^ A zwitterionic S-MGB, S-MGB-241, **36**, was discovered that is selectively potent against *A. castellanii* with an IC_50_ of 6.6 µM in a trophocidal assay, and non-cytotoxic (*IC_50 _*> 100 µM) against HEK293 cells.

S-MGB-241, **36**, has been shown to stabilize gDNA using a thermal shift assay, and also bind to a short AT-rich dsDNA oligomer using native mass spectrometry, albeit weakly in both cases. This is in line with the lower relative potency of S-MGB-241, **36**, compared to other S-MGBs that are effective against different pathogens and have concomitantly greater DNA binding. However, a different mechanism of action not involving DNA cannot be ruled out entirely.

## Methods

### Anti-acanthamoeba trophocidal assay

The *Acanthamoeba castellanii* Neff strain was donated by Keith Vickerman. The microtiter plate alamarBlue^™^, resazurin assay was carried out as previously described.^19^ The compounds were tested over a range of 10-fold dilution concentrations in triplicate against *Acanthamoeba castellanii* (4 × 10^4^ seeding density), then the plates were incubated at 23°C for 96 hours. Six hours before the end of the incubation period, 10 µL of alamarBlue^™^ reagent (BioRad, UK) was aseptically added to all the wells except the blank. Afterwards, the plate was incubated for 6 hours at 23°C in dark conditions. Controls were included in each plate, consisting of PG blank, trophozoites with medium alone, trophozoites with the highest concentration of solvent (DMSO), PG medium with the highest concentration of S-MGB alone and PG medium alone with alamarBlue. The final volume for all wells was 100 µL. Finally, the absorbance was then read on a Spectromax (Molecular Devices, USA) spectrophotometer at OD570 and OD600.

### Antibacterial assay

The MIC against *S. aureus* ATCC 43300 and *E. faecalis* ATCC 51299 was measured by making two-fold serial dilutions of the samples into a 96-well non-binding surface plate (Corning #3640). Bacteria were cultured in CAMHB overnight at 37°C, diluted 40-fold and incubated for a further 1.5–3 h at 37°C. The resultant mid-log phase cultures were diluted and added to each well of the compound containing plates, giving a cell density of 5 × 10^5^ cfu/mL, measured by absorbance at 600 nm (OD_600_) and a final compound concentration range of 50–0.0195 μM. All plates were covered and incubated at 37°C for 18 h without shaking. Inhibition of bacterial growth was determined by OD_600_, using a Tecan Infinite M Nano plate reader. The percentage of growth inhibition was calculated for each well, correcting for background using a negative control (medium only) and a positive control (bacteria without inhibitors) on the same plate. The MIC was determined as the lowest concentration at which the growth was fully inhibited, defined by an inhibition 80%. Each MIC determination was carried out in triplicate on separate days.

### *Anti-*Trypanosoma *assay*

Bloodstream form *T. b. brucei* (Lister 427) was cultured in HMI-11 medium (Gibco) supplemented with 10% heat inactivated FBS (Gibco), at 37°C in a humidified 5% CO_2_ environment. EC_50_ values against *T. b. brucei* were determined by an *in vitro* alamarBlue^™^ assay. *T. b. brucei* parasites (2 × 10^4^ cells per mL) were seeded into serial dilutions of the test compounds to a final volume of 200 μL and incubated for 48 h, after which 20 μL of 0.49 mM resazurin dye (Sigma-Aldrich) was added and cells were incubated for a further 24 h. The reduction of resazurin was measured using a fluorimeter (FLUOstar Optima, BMG Labtech) at 544 nm excitation and 590 nm emission wavelengths. MIC values were identified as the minimum concentration required to inhibit 80% of growth. All experiments were carried out on at least three independent occasions.

### HEK293 cytotoxicity

Human embryonic kidney cells (HEK293) were obtained from Sigma-Aldrich, UK. The cells were seeded in 75 cm^2^ vented flasks at a concentration of 1.5 × 10^4^ cells/well in DMEM, pH 7.4, and grown in a humidified incubator at 37°C in the presence of 5% CO_2_. The DMEM was supplemented with 10% FBS, 1% penicillin/streptomycin, 2 mM L-glutamine, 1 mM sodium pyruvate and either galactose (4.5 g/L) or glucose (4.5 g/L) (all consumables Sigma-Aldrich, UK). Cells were grown to 80% confluence and passaged using 1X TrypLe^™^ express enzyme (Thermo Fisher Scientific, UK)

For viability determination, HEK293 cells were seeded at a concentration of 1500 cells/well (50 µL) in black 96-well half-area clear bottomed plates (Greiner, UK). Cells were allowed to attach overnight then were exposed to a final concentration of 12.5, 25, 50 and 100 µM S-MGB-241 in a total volume of 60 µL per well (stock S-MGB-241 was dissolved in DMSO at a concentration of 10 mM). Cells in the absence of compound were termed control wells and represented 100% viability. Complete kill was determined by the addition of 0.1% Triton X. Cell viability was assessed after 24 hours of incubation at 37°C (humidified, 5% CO_2_) using 10% v/v PrestoBlue^TM^ HS Cell Viability reagent (Sigma-Aldrich, UK) according to manufacturer’s instructions. The resulting reduction of this resazurin dye (Presto Blue^™^) was quantified using a Hidex Sense multimode plate reader (Lablogic, UK and Ireland) in fluorescent mode with excitation and emission wavelengths of 535/20 and 595/10 nm, respectively.

The viability of the treated samples was expressed as a percentage of the control wells containing DMSO (vehicle). Statistical analysis was performed using the mean ± standard error (SE) values derived from three independent experiments.

Data were plotted using Graph Pad Prism Software: Graph Pad Prism version 9.00 for Windows (GraphPad Software, La Jolla CA, USA: www.graphpad.com)

### UV−vis DNA thermal melting experiments

Salmon genomic DNA (gDNA; D1626, Sigma-Aldrich) at 1 mg/mL in 1 mM phosphate buffer (pH 7.4) containing 0.27 mM KCl and 13.7 mM NaCl (P4417, Sigma-Aldrich) was annealed at 90°C for 10 min and left to cool to room temperature. S-MGBs at 10 mM in DMSO were diluted with the same phosphate buffer to yield a single sample with 10 μM S-MGB and 0.02 mg/mL gDNA in 1 mM phosphate buffer containing 0.27 mM KCl and 13.7 mM NaCl. Control samples containing only S-MGB or gDNA were prepared, respectively. Samples were melted at a rate of 0.5°C/min from 45 to 90°C with spectra recorded at 260 nm on a UV-1900 UV-vis spectrophotometer fitted with a Peltier temperature controller (Shimadzhu) using LabSolutions (Tm Analysis) software. The melting temperatures (*T*_m_s) of the S-MGB:DNA complexes were determined by fitting a sigmoidal function using a Boltzmann distribution in OriginPro.

### Native mass spectrometry

For DNA sample preparation, DNA oligonucleotide sequence 5′-CGCATATATGCG-3′ was purchased in lyophilized form from Alpha DNA (Canada) and used without further purification, purity assessed by NMR. Then 100 μM stock solutions of DNA were prepared with 150 mM ammonium acetate solution (Fisher Scientific, Loughborough, Leicestershire, UK) and 2 mM potassium chloride solution (Fisher Scientific, Loughborough, Leicestershire, UK). This solution was annealed at 90°C for 10 minutes and allowed to cool to room temperature. Next, 10 mM S-MGB stock in 100% DMSO (Sigma-Aldrich, St Louis, MO, USA) were diluted to 1 mM S-MGB solution with 150 mM ammonium acetate. Final samples were prepared from this solution to yield final concentrations of 9 μM DNA, 100 μM KCl and 100 μM s-MGB, 1% DMSO. DNA solutions containing no S-MGB included 1% DMSO and were used as controls.

When taking the mass spectrometry measurements, native mass spectrometry experiments were carried out on a Synapt G2Si instrument (Waters, Manchester, UK) with a nanoelectrospray ionization source (nESI). Mass calibration was performed by a separate infusion of NaI cluster ions. Solutions were ionized from a thin-walled borosilicate glass capillary (i.d. 0.78 mm, o.d. 1.0 mm, (Sutter Instrument Co., Novato, CA, USA) pulled in-house to nESI tip with a Flaming/Brown micropipette puller (Sutter Instrument Co., Novato, CA, USA). A negative potential in range of 1.0–1.2 kV was applied to the solution via a thin platinum wire (diameter 0.125 mm, Goodfellow, Huntingdon, UK). The following non-default instrument parameters were used for the DNA: S-MGB-241 complex: capillary voltage 1.4 kV, sample cone voltage 100 V, source offset 110 V, source temperature 40°C, trap collision energy 3.0 (V), trap gas 3 mL/min. For DNA with no MGB present, the following parameters were changed: capillary voltage 1.0 kV, sample cone voltage 80 V, source offset 95 V and trap gas 4.0 mL/min. Data were processed using Masslynx v.4.2 and OriginPro v.2021, and figures were produced using ChemDraw.

## Results and discussion

### Chemistry

**Figure 2.**
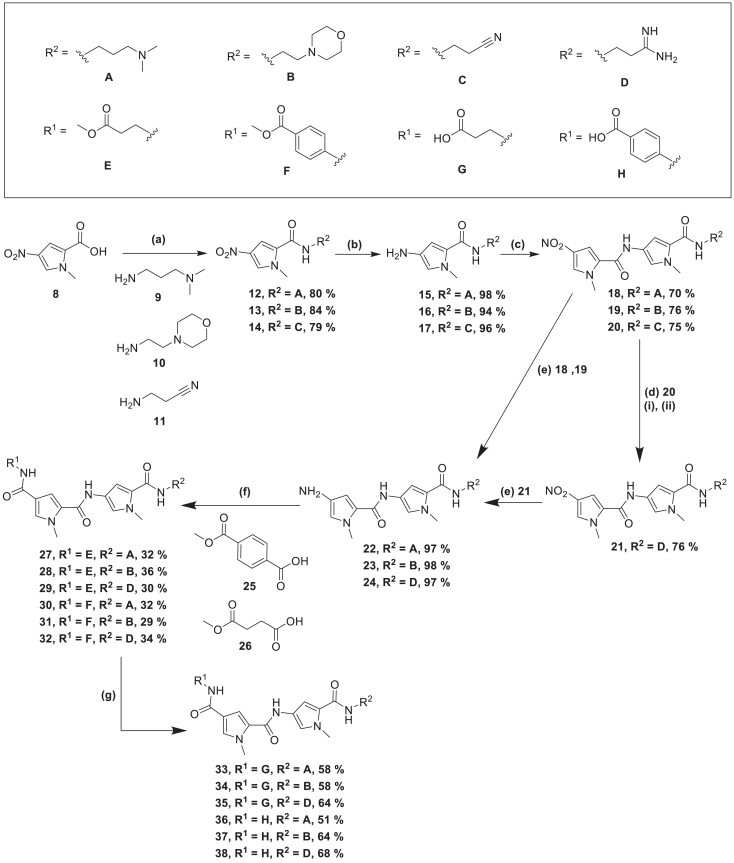
Synthetic route used to generate final S-MGB compounds **27–38.** Reagents and conditions: (a) HBTU, amine **9**, **10** or **11,** DMF. (b) (i) Pd/C, MeOH, 2 h. (c) HBTU, DMF. (d) (i) **20**, HCl (gas), EtOH, −60°C, 1.5 h, RT, 14 h. (ii) NH_3_, MeOH, 55°C, 6 h. (e) Pd/C, MeOH, amine **18**, **19** or **21**, 4 h. (f) HBTU, with acid **25**, or **26** when **R^2^ = A**, **B** or **D**, DMF, RT, overnight. (g) **27**–**32**, LiOH, H_2_O, MeOH, 80–100°C.

A panel of 12 S-MGBs was synthesized to investigate the biological activity of zwitterionic S-MGBs. The methodology associated with the synthesis can be found in the Supplementary Information. The S-MGBs were constructed from previously synthesized dimers **22, 23** and **24** (Figure 2).^20,21^ Briefly, an HBTU-mediated amide coupling of the appropriate amine (**9, 10** or **11**) with nitro pyrrole carboxylic acid, **8**, provided **12**, **13** and **14**. This was followed by hydrogenation of the nitro group to an amine, followed directly by another HBTU-mediated coupling with nitro pyrrole carboxylic acid, **8**, to yield nitro dimers **18**, **19** and **20**. A Pinner reaction of nitro dimer **20** provided access to amidine dimer **21**. Methyl ester containing S-MGBs **27**–**32**, were obtained through an HBTU-mediated amide coupling of carboxylic acids **25** or **26** with the appropriate amino dimers **22**–**24**, which were in turn obtained via hydrogenation using Pd/C from the corresponding nitro compounds (**18**, **19** and **21**). All methyl ester compounds were subsequently hydrolysed using lithium hydroxide to obtain the corresponding zwitterionic S-MGBs **33**–**38** in moderate yields (51%–68%) and >95% purity.

### Biological evaluation

The set of 12 S-MGBs was evaluated against *Acanthamoeba castellanii* (Neff strain) as an indication of anti-amoeba activity, with miltefosine as a positive control. Additionally, the compounds were screened against *Trypanosoma brucei brucei* (Lister 427), *Staphylococcus aureus* (ATCC 43300) and *Escherichia coli* (ATCC 25922) (Table 1). Dose–response curves against *Acanthamoeba castellanii* (Neff strain) can be found in the Supplementary Information (Figure S1, available as Supplementary data at *JAC* Online).

**Table 1.**
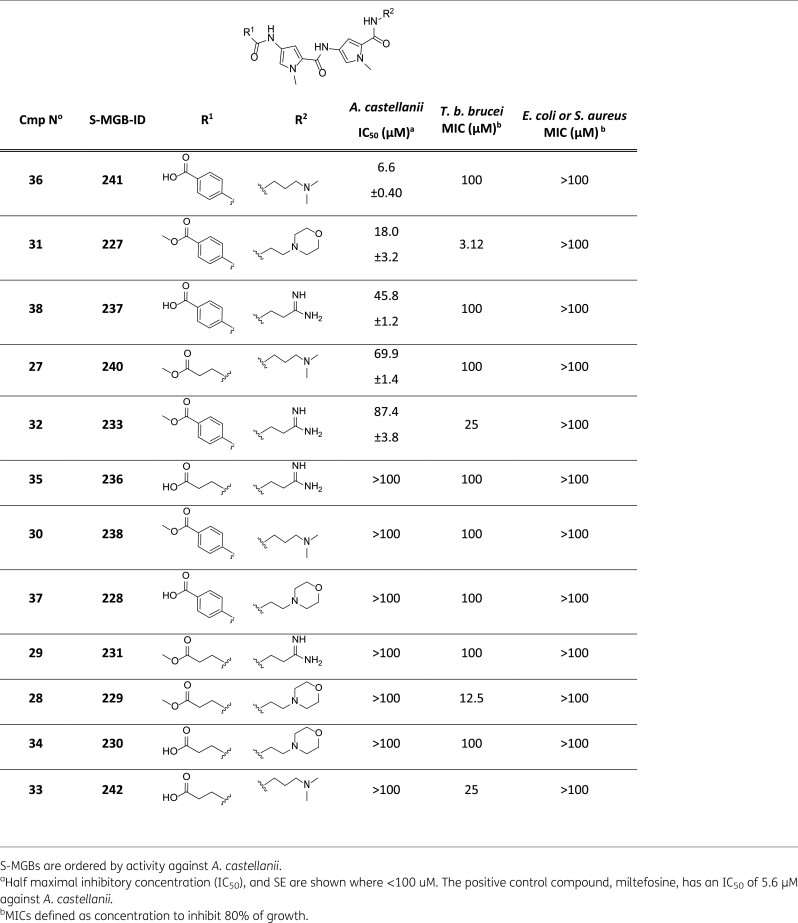
Activity profiles of 12 tested S-MGBs against *Acanthamoeba castellanii*, *Trypanosoma brucei*, *Staphylococcus aureus* and *Escherichia coli*.

S-MGB-241, **36**, is the most active compound against *A. castellanii* (IC_50_ of 6.6 µM), and the most selective to this organism over the others tested. The zwitterionic nature of this S-MGB, and thus lower logP, may be significant in the observed potency towards *Acanthamoeba castellanii*, as a comparison of S-MGB-241, **36**, with its methyl ester analogue, S-MGB-238, **30**, reveals the latter to have no measurable activity against *A. castellanii*. However, a pair-wise comparison across all carboxylic acid and methyl ester pairs does not consistently maintain this trend, which suggests that logP and the zwitter ionic nature are not sufficient explanatory factors. It is also possible that there is a confounding effect from the tail group. Indeed, there does not appear to be an obvious trend when considering only the basicity of the tail group either as the dimethyl amino tail, with intermediate basicity, led to the most active compound, **36**. None-the-less, S-MGB-241, **36**, is the first zwitterionic S-MGB with notable antimicrobial activity that has been identified from the S-MGB platform.

S-MGB-227, **31**, exhibits moderate potency for *A. castellanii* (IC_50_ of 18.01 µM), and activity against *Trypanosoma brucei* (MIC 3.12 µM). The amidine containing analogue of S-MGB-227, **31**, S-MGB-233, **32**, also exhibited moderate activity towards *Trypanosoma brucei,* but was less potent than its morpholine analogue against both parasites. None of the compounds were found to have measurable activity against either of the bacterial strains, *E. coli* or *S. aureus*.

The cytotoxicity of S-MGB-241, **36**, was evaluated against HEK293 cells, using both galactose and glucose supplementation. S-MGB-241, **36**, was determined to have an IC_50_ > 100 µM (Figure S2) in both cytotoxicity models, providing a satisfactory selectivity index (>15).

The kinetics of S-MGB-241 inhibition of *A. castellanii* was further investigated by truncating treatment duration to 24 h. S-MGB was found to inhibit AlmarBlue reduction by *Acanthamoeba* in a dose dependent manner with maximal effect noted at concentrations above 50 µM (Figure S3). Microscopy confirmed that the cultures treated with S-MGB-241 had fewer Acanthamoeba present. Acanthamoeba present at 24 hours exhibited morphological changes including rounding and less distinct membranes. These morphological changes were maintained 144 hours after removal of S-MGB-241, indicating that the effects of S-MGB-241 are long lived and potentially cidal (Figure S4).

These biological activity data identified S-MGB-241, **36,** as a compound worthy of further investigation due to its selective potency against *A. castellanii* and comparable activity to the clinically used compound, miltefosine.

### Biophysical studies

Other S-MGBs have been shown to bind to double stranded DNA (dsDNA) using a variety of methods, including thermal shift analysis of genomic DNA (gDNA) and native mass spectrometry using short AT-rich dsDNA oligomers.^11,15^ Both techniques were employed on S-MGB-241, **36**, to provide evidence of DNA binding.

**Figure 3.**
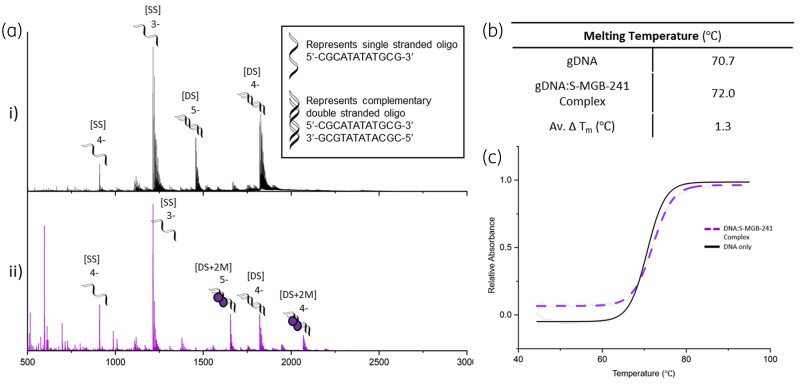
(a) Characterization of S-MGB-241, **36**, binding to double stranded DNA as a dimer by nMS. nESI-MS of DNA sequence 5′-CGCATATATGCG-3*’* (9 μM DNA, 100 μM KCl, 1% DMSO) sprayed from ammonium acetate (150 mM, pH 7) in the absence (i) or presence (ii) of 100 μM S-MGB. (i) Single stranded DNA (denoted [SS]) is present in charge states 4*-* and 3-, and double stranded DNA (denoted [DS]) is present in charge states 5- and 4-. (ii) [SS] is present in charge states 3- and 4-. [DS] is present in charge state 4-. Each bound [DS] molecule is seen to bind 2xS-MGB-241 molecules (denoted [DS + 2 M]) and is present in charge states 5- and 4-. (b) and (c) Thermal melt analysis data of S-MGB-241, **36** bound to gDNA, including exemplar melt curve from one experimental repeat, fitted with a Boltzmann distribution. Additional details of native mass spectrometry and thermal melt analysis are presented in the Supplementary Information (Tables S1–S4). This figure appears in colour in the online version of *JAC* and in black and white in the print version of *JAC*.

Native mass spectrometry was carried out using a short, self-complementary DNA oligo with an AT-rich binding site: 5′-CGCATATATGCG-3′ (Figure 3). The choice of dsDNA oligo, containing an AT-rich binding site, is based on our previous studies on S-MGBs showing that this oligo is good for evaluating their binding due to similar binding preferences to distamycin, the template from which S-MGBs are derived. Analysis of the native MS spectrum of dsDNA oligo 5′-CGCATATATGCG-3′ in the presence of S-MGB-241, **36**, provides conclusive evidence that the S-MGB binds as a dimer [DS + 2 M], in charge states 5- and 4- (Figure 3). No evidence of S-MGB-241, **36**, bound as a monomer [DS + 1 M] was observed. There is also a strong *m/z* peak corresponding to the free dsDNA oligo in the S-MGB-241-treated experiment. This is perhaps indicative of weaker binding compared to S-MGBs that have been explored in other studies, for which free dsDNA is not strongly observed.^11,15^

The DNA thermal shift of S-MGB-241, **36**, using a model gDNA extracted from salmon, showed a statistically significant, but small increase in melting temperature, Δ*T*_m_ = 1.3 ± 0.1°C (SE; Figure 2b). Under these experimental conditions we have observed potent (<1 µM) antibacterial S-MGBs to have Δ*T*_m_ values in the range of 1 to >15°C, and therefore S-MGB-241, **36**, would be considered a weakly binding compound.^11,15^

### Conclusion

Herein 12 previously unpublished S-MGBs have been synthesized and evaluated for biological activity against *A. castellanii*, *T. b. brucei*, *S. aureus* and *E. coli*. S-MGB-241, **36**, was identified as a selective anti-*Acanthamoeba* agent with an IC_50_ of 6.6 µM against *A. castellanii*, comparable in potency to the clinically used miltefosine (5.8 µM).

Although it has been used clinically as an adjunct therapy for *AK*, miltefosine, is liable to induce toxic side effects due to its membrane permeabilizing mechanism of action. Indeed, none of the existing clinical options are particularly effective, and most suffer from adverse side effects. Therefore, S-MGB-241, **36**, is a valuable ‘hit’ compound for future optimization.

Both a thermal shift assay and native mass spectrometry confirmed that S-MGB-241, **36**, binds to dsDNA, albeit weakly compared to S-MGBs from other anti-infective drug discovery programmes. For example, MGB-BP-3 has sub-micromolar potency against various Gram-positive bacteria and has Δ*T*_m_ >15°C in the same DNA thermal shift assay, compared to a Δ*T*_m_ = 1°C for S-MGB-241, **36**.^15^ Similarly, S-MGB-364, a compound with an MIC_99_ of 1.56 µM against *Mycobacterium tuberculosis* (H37Rv-GFP), has a Δ*T*_m_ = 16°C.^11,22^ In line with their stronger DNA binding, both MGB-BP-3 and S-MGB-364 show limited evidence of unbound dsDNA oligo in similar native mass spectrometry experiments as carried out herein with S-MGB-241, **36**.^11,15^

The potency of MGB-BP-3 was also measured against *A. castellanii* (Figure S5), which had an IC_50_ of ∼50 µM, about 8-fold higher than S-MGB-241. This further suggests that DNA binding alone does not account for potency. Indeed, potency for antimicrobials that target an intracellular mechanism is governed by both target engagement and intracellular accumulation. Therefore, we suggest that S-MGB-241’s weak DNA binding is compensated by a greater intracellular accumulation to account for a higher potency against *A. castellanii* compared to MGB-BP-3. Improvements in potency of S-MGB-241 could thus be achieved by enhancing its DNA binding without compromising its favourable intracellular accumulation. This is consistent with findings from across our S-MGB drug discovery platform, where selectivity appears to be driven by differential accumulation in pathogen or mammalian cells rather than through differences in target engagement, although DNA binding is a minimal characteristic for potency.^21^

The shorter structure of S-MGB-241, **36**, in comparison to some other S-MGBs is the likely origin of its comparatively weaker DNA binding. Indeed, we have previously observed a series of ‘truncated’ S-MGBs with selective anti-parasitic properties, lacked potent antibacterial activity.^18^ Both studies suggest that shorter S-MGBs possess weaker binding, but may also contribute to selective activity towards parasites. Therefore, future optimization strategies for *A. castellanii* potency could seek to increase DNA binding without altering the length of the S-MGB.

In conclusion, S-MGB-241, **36**, has been identified as a zwitterionic S-MGB that is selectively active against *A. castellanii*, and with comparable potency to miltefosine, which is used clinically in the treatment of Acanthamoeba infections. The zwitterionic structure of S-MGB-241, **36**, may pose an issue for downstream drug development efforts; however, this ‘hit’ compound provides a starting point from which to optimize the structure, with a focus on improving DNA binding, to increase potency.

## Supplementary Material

dkae221_Supplementary_Data

## Supplementary data

Figures S1 to S5 and Tables S1 to S4 are available as Supplementary data at *JAC* Online.

## References

[1] Wang Y , Jiang L, Zhao Y et al Biological characteristics and pathogenicity of Acanthamoeba. Front Microbiol 2023; 14: 1147077. 10.3389/fmicb.2023.1147077 37089530 PMC10113681

[2] Ahmed U , Anwar A, Ong S-K et al Applications of medicinal chemistry for drug discovery against Acanthamoeba infections. Med Res Rev 2022; 42: 462–512. 10.1002/med.21851 34472107

[3] Azzopardi M , Chong YJ, Ng B et al Diagnosis of *Acanthamoeba* keratitis: past, present and future. Diagnostics 2023; 13: 2655. 10.3390/diagnostics13162655 37627913 PMC10453105

[4] Zhang H , Cheng X. Various brain-eating amoebae: the protozoa, the pathogenesis, and the disease. Front Med 2021; 15: 842–66. 10.1007/s11684-021-0865-2 34825341

[5] Elsheikha HM , Siddiqui R, Khan NA. Drug discovery against *Acanthamoeba* infections: present knowledge and unmet needs. Pathogens 2020; 9: 405. 10.3390/pathogens9050405 32456110 PMC7281112

[6] Taravaud A , Fechtali-Moute Z, Loiseau PM et al Drugs used for the treatment of cerebral and disseminated infections caused by free-living amoebae. Clin Transl Sci 2021; 14: 791–805. 10.1111/cts.12955 33650319 PMC8212752

[7] Juárez MM , Tártara LI, Cid AG et al Acanthamoeba in the eye, can the parasite hide even more? Latest developments on the disease. Cont Lens Anterior Eye 2017; 41: 245–51. 10.1016/j.clae.2017.12.017 29273391

[8] Dart JKG , Saw VPJ, Kilvington S. *Acanthamoeba* keratitis: diagnosis and treatment update. Am J Ophthalmol 2009; 148: 487–99. 10.1016/j.ajo.2009.06.009 19660733

[9] Thulasi P , Saeed HN, Rapuano CJ et al Oral miltefosine as salvage therapy for refractory *Acanthamoeba* keratitis. Am J Ophthalmol 2021; 223: 75–82. 10.1016/j.ajo.2020.09.048 33045218

[10] Kieswetter NS , Ozturk M, Hlaka L et al Intranasally administered S-MGB-364 displays antitubercular activity and modulates the host immune response to *Mycobacterium tuberculosis* infection. J Antimicrob Chemother 2022; 77: 1061–71. 10.1093/jac/dkac001 35084027 PMC8969509

[11] Giordani F , Khalaf AI, Gillingwater K et al Novel minor groove binders cure animal African trypanosomiasis in an in vivo mouse model. J Med Chem 2019; 62: 3021–35. 10.1021/acs.jmedchem.8b01847 30763102

[12] Scott FJ , Nichol RJO, Khalaf AI et al An evaluation of minor groove binders as anti-fungal and anti-mycobacterial therapeutics. Eur J Med Chem 2017; 136: 561–72. 10.1016/j.ejmech.2017.05.039 28544982

[13] Khalaf AI , Anthony N, Breen D et al Amide isosteres in structure-activity studies of antibacterial minor groove binders. Eur J Med Chem 2011; 46: 5343–55. 10.1016/j.ejmech.2011.08.035 21908079

[14] Hind C , Clifford M, Woolley C et al Insights into the spectrum of activity and mechanism of action of MGB-BP-3. ACS Infect Dis 2022; 8: 2552–63. 10.1021/acsinfecdis.2c00445 36444998 PMC9745797

[15] Suckling CJ , Hunter IS, Scott FJ. Multitargeted anti-infective drugs: resilience to resistance in the antimicrobial resistance era. Future Drug Discovery 2022; 4: FDD73. 10.4155/fdd-2022-0001 35600289 PMC9112235

[16] Kerr L , Browning DF, Lemonidis K et al Novel antibiotic mode of action by repression of promoter isomerisation. BioRxiv 2021: 12.31.424950. 10.1101/2020.12.31.424950

[17] Alniss HY , Khan NA, Boghossian A et al Synthesis and evaluation of novel DNA minor groove binders as antiamoebic agents. Antibiotics 2022; 11: 935. 10.3390/antibiotics11070935 35884189 PMC9312114

[18] Brooke DP , McGee LMC, Giordani F et al Truncated S-MGBs: towards a parasite-specific and low aggregation chemotype. RSC Med Chem 2021; 12: 1391–401. 10.1039/d1md00110h 34447938 PMC8372214

[19] McBride J , Ingram PR, Henriquez FL et al Development of colorimetric microtiter plate assay for assessment of antimicrobials against acanthamoeba. J Clin Microbiol 2005; 43: 629–34. 10.1128/JCM.43.2.629-634.2005 15695656 PMC548097

[20] Khalaf AI , Waigh RD, Drummond AJ et al Distamycin analogues with enhanced lipophilicity: synthesis and antimicrobial activity. J Med Chem 2004; 47: 2133–56. 10.1021/jm031089x 15056010

[21] Tentellino C , Tipping WJ, McGee LMC et al Ratiometric imaging of minor groove binders in mammalian cells using Raman microscopy. RSC Chem Bio 2022; 3: 1403–15. 10.1039/d2cb00159d 36544571 PMC9709774

[22] Hlaka L , Rosslee M-J, Ozturk M et al Evaluation of minor groove binders (MGBs) as novel anti-mycobacterial agents and the effect of using non-ionic surfactant vesicles as a delivery system to improve their efficacy. J Antimicrob Chemother 2017; 72: 3334–41. 10.1093/jac/dkx326 28961913 PMC5890746

