# Supplementary material for: Strathclyde minor groove binders (S-MGBs) with activity against Acanthamoeba castellanii
Source: J Antimicrob Chemother. 2024 Jul 9;79(9):2251–8. doi: 10.1093/jac/dkae221 (PMC11368431; doi:10.1093/jac/dkae221)
Supplement: dkae221_Supplementary_Data [file dkae221_supplementary_data.docx]

**Strathclyde Minor Groove Binders (S-MGBs) with activity against *Acanthamoeba castellanii:* Supplementary Information**

Leah M. C. McGee,^1^ Alemao G. Carpinteyro Sanchez,^2^ Marina Perieteanu,^1^ Kaveh Eskandari,^1^ Yan Bian,^1^ Logan MacKie,^2^ Louise Young,^2^ Rebecca Beveridge,^1^ Colin J. Suckling,^1^ Craig W. Roberts^2^ and Fraser J. Scott^1^

^1^Department of Pure and Applied Chemistry, University of Strathclyde, Glasgow UK

^2^Strathclyde Institute of Pharmacy and Biomedical Sciences, University of Strathclyde, Glasgow UK

Email: [fraser.j.scott@strath.ac.uk](mailto:fraser.j.scott@strath.ac.uk)

**Table of Contents**

1. IC_50_ Plots for S-MGBs against *Acanthamoeba castellanii,* page 2

2. Cytotoxicity of S-MGB-241 against HEK293 cells, page 3

3. 24-hour IC_50_ Plot of S-MGB-241 against *Acanthamoeba castellanii,* page 3

4. Morphological changes induced by S-MGB-241 at 24 hours treatment, page 4

5. 96-hour IC_50_ Plot of MGB-BP-3 against *Acanthamoeba castellanii,* page 4

6. Synthetic Chemistry, page 5

6.1 General Methods, page 5

6.2 General Procedure for Novel S-MGBs, page 5

6.3 Characterisation Data for Novel S-MGBs, page 6

6.4 Biophysical Studies of S-MGB-241, page 9

7. References, page 10

**1. IC_50_ Plots for S-MGBs against *Acanthamoeba castellanii***


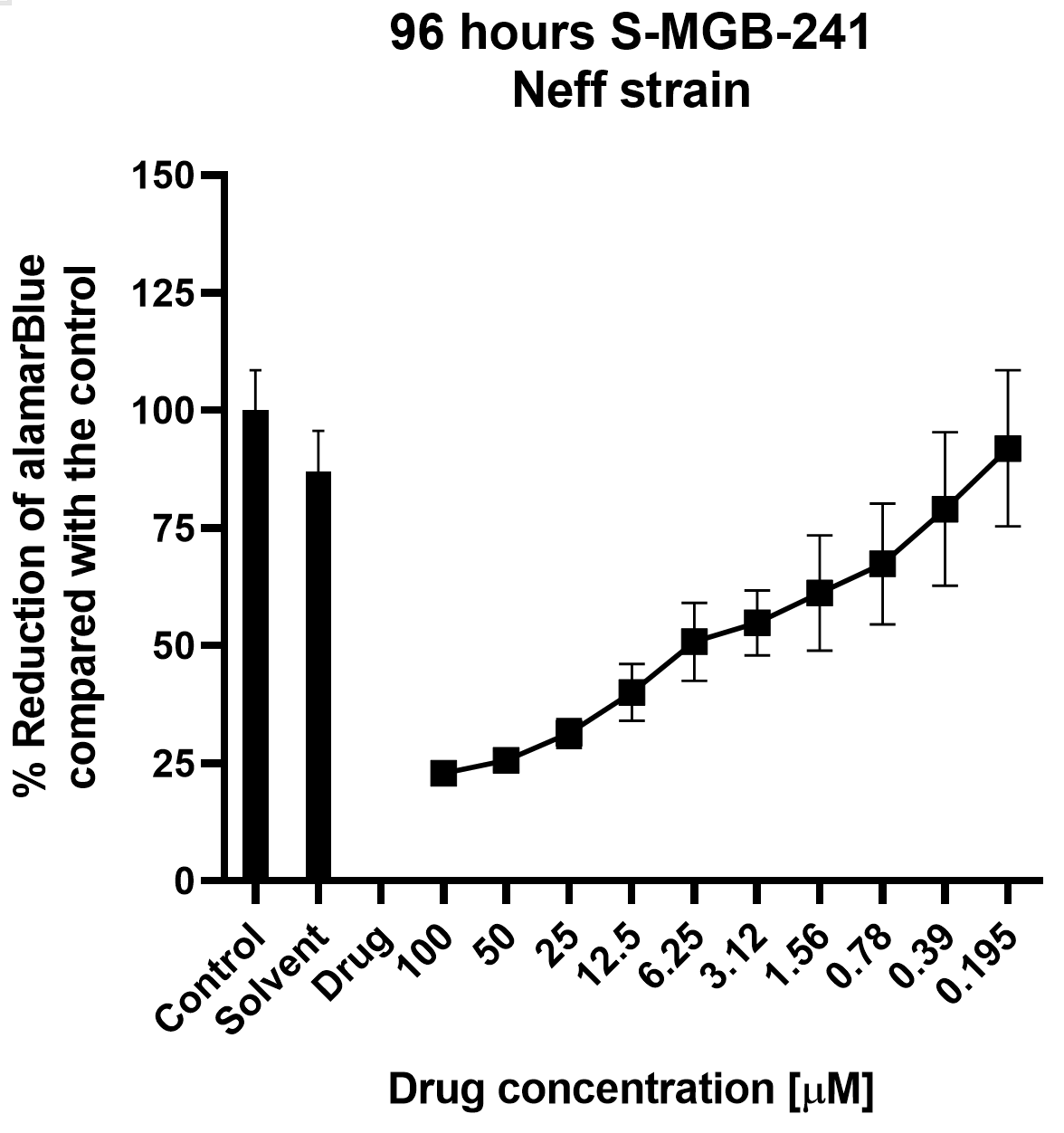

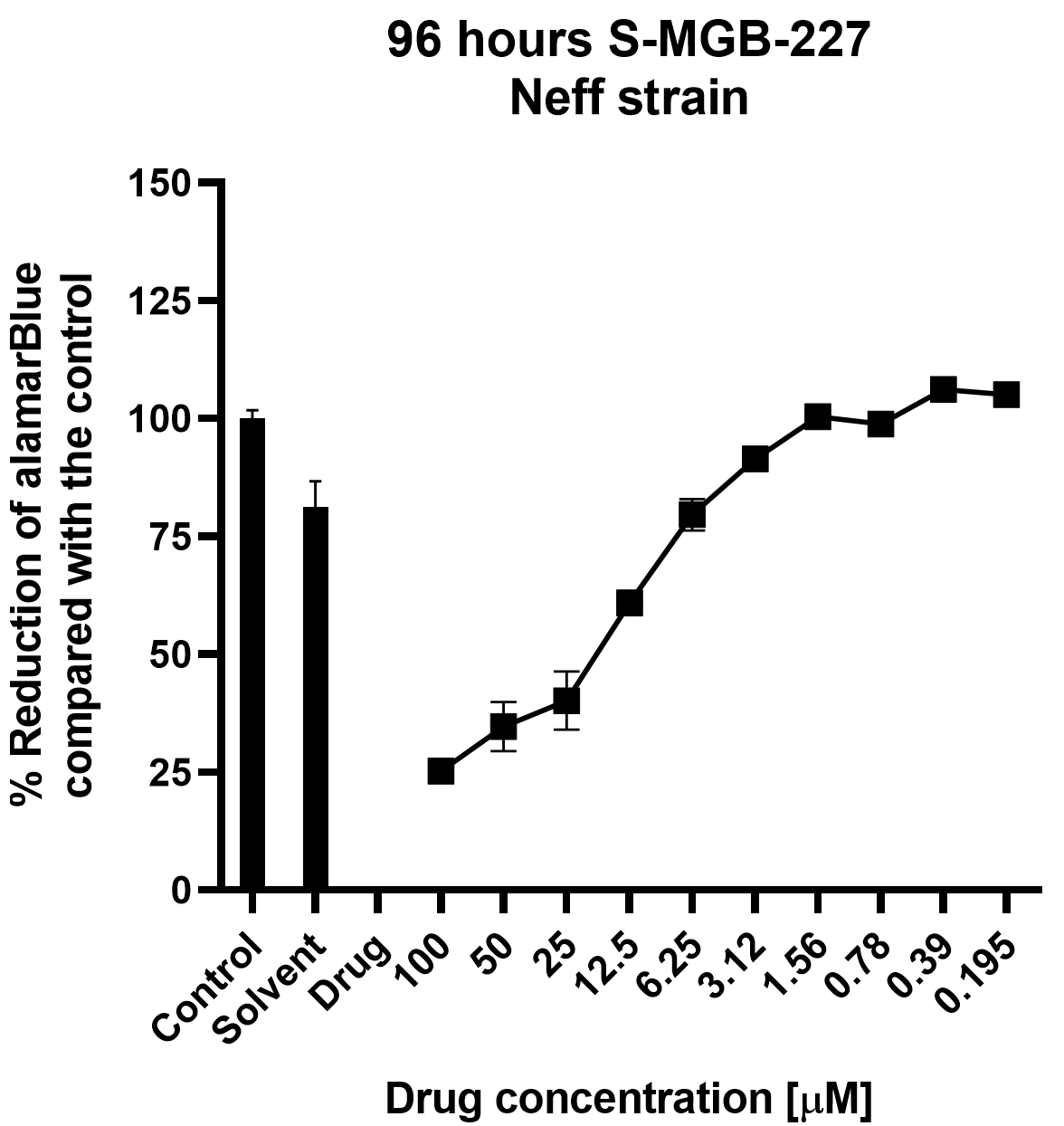

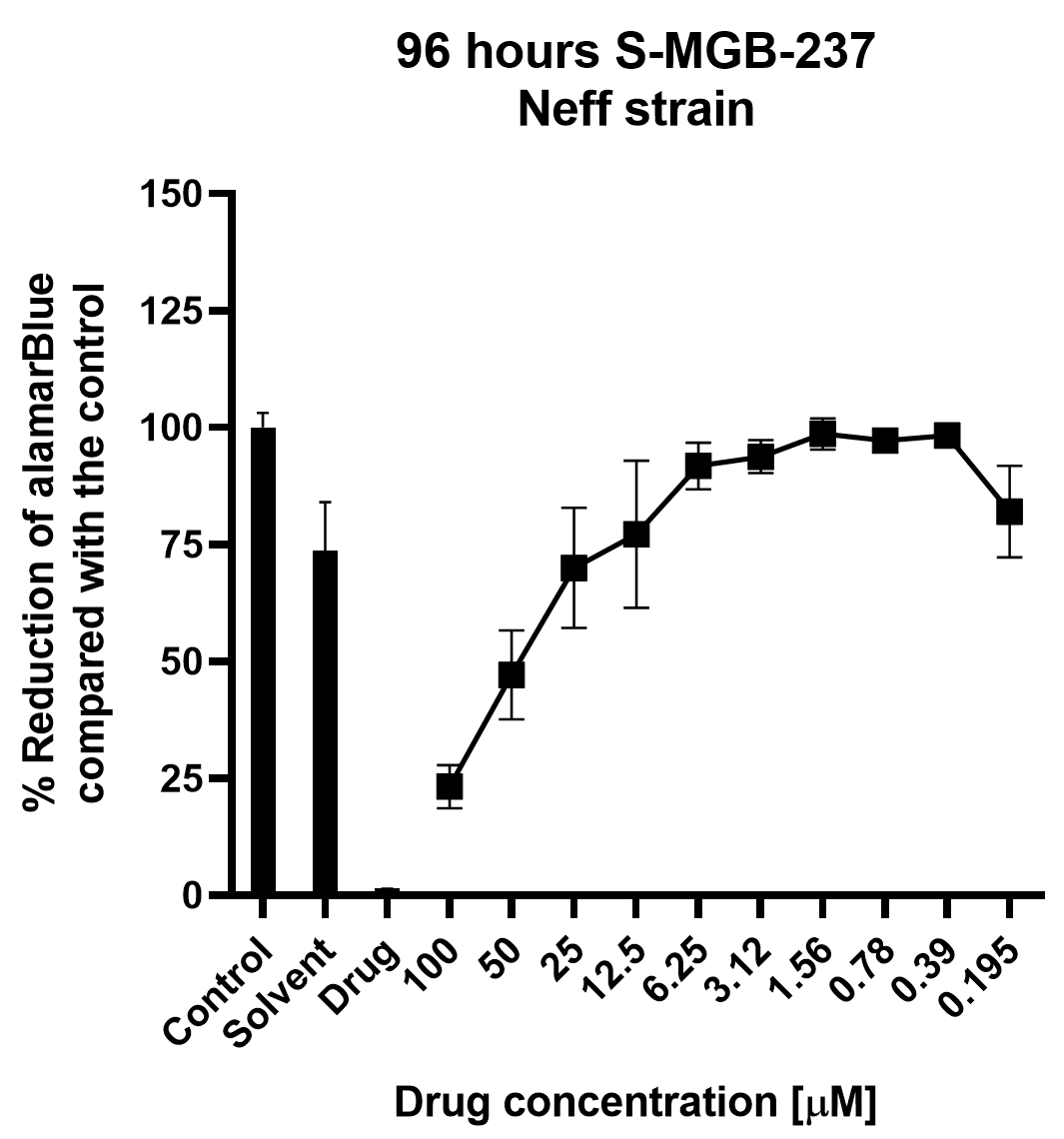


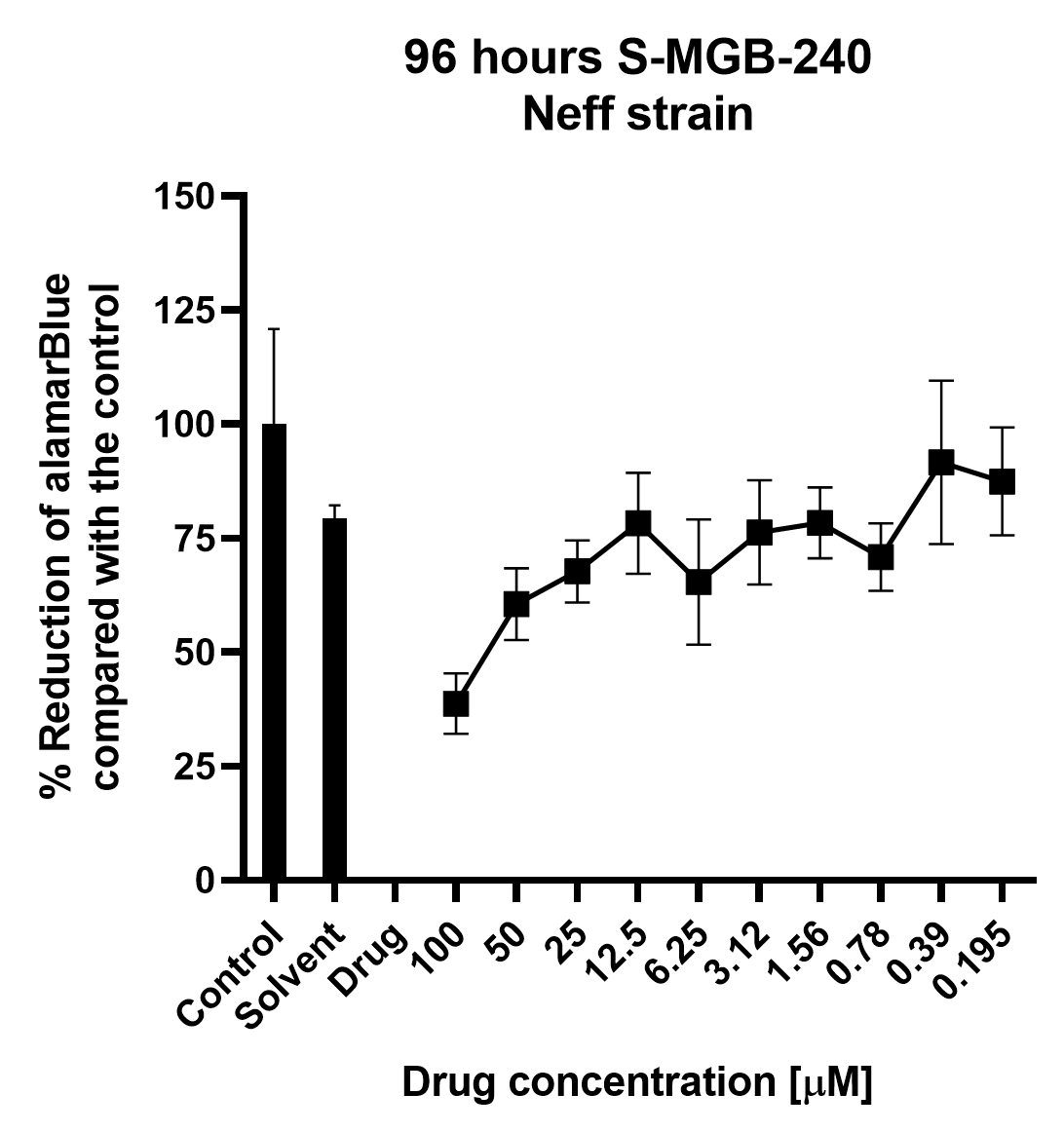

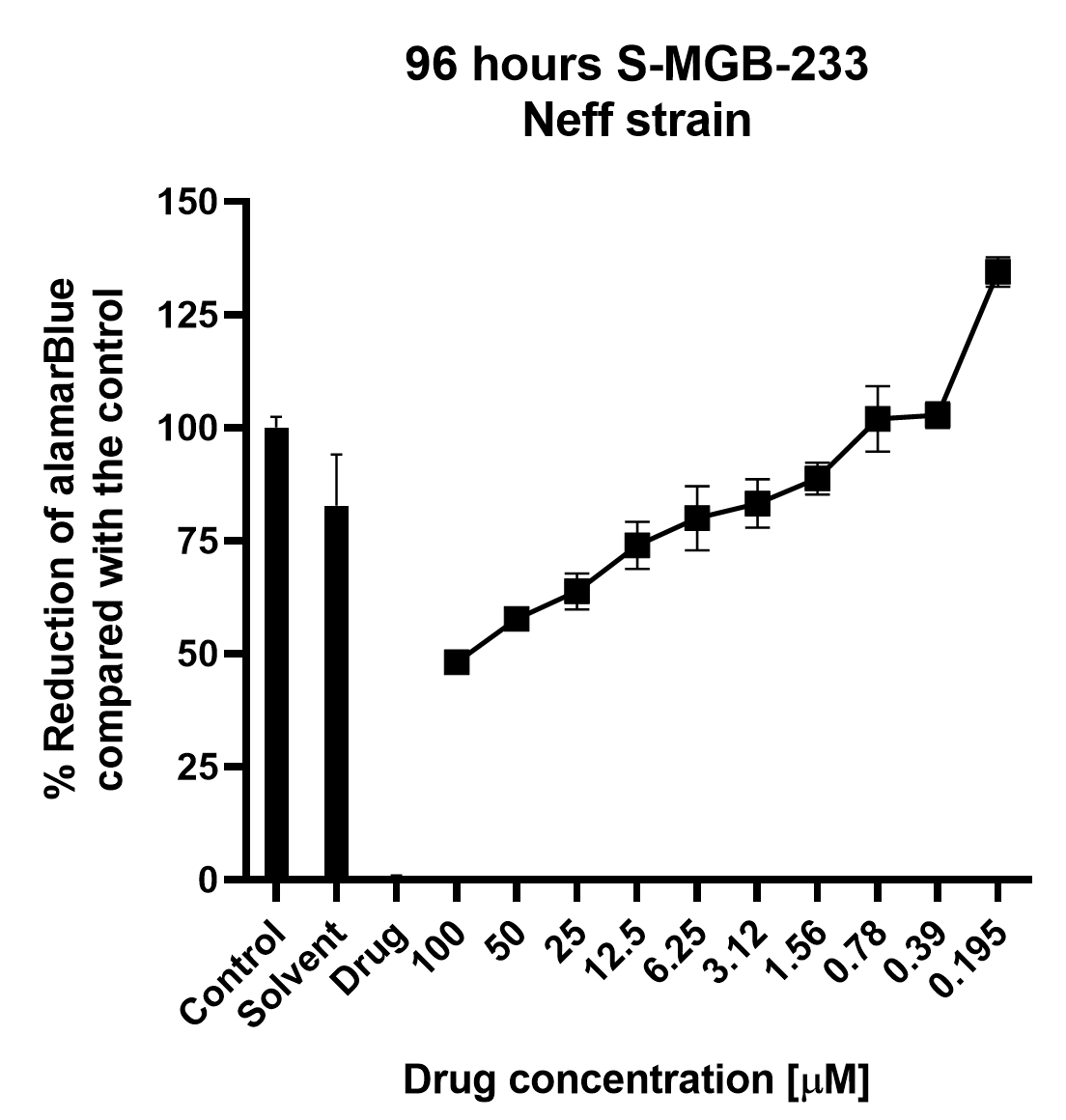

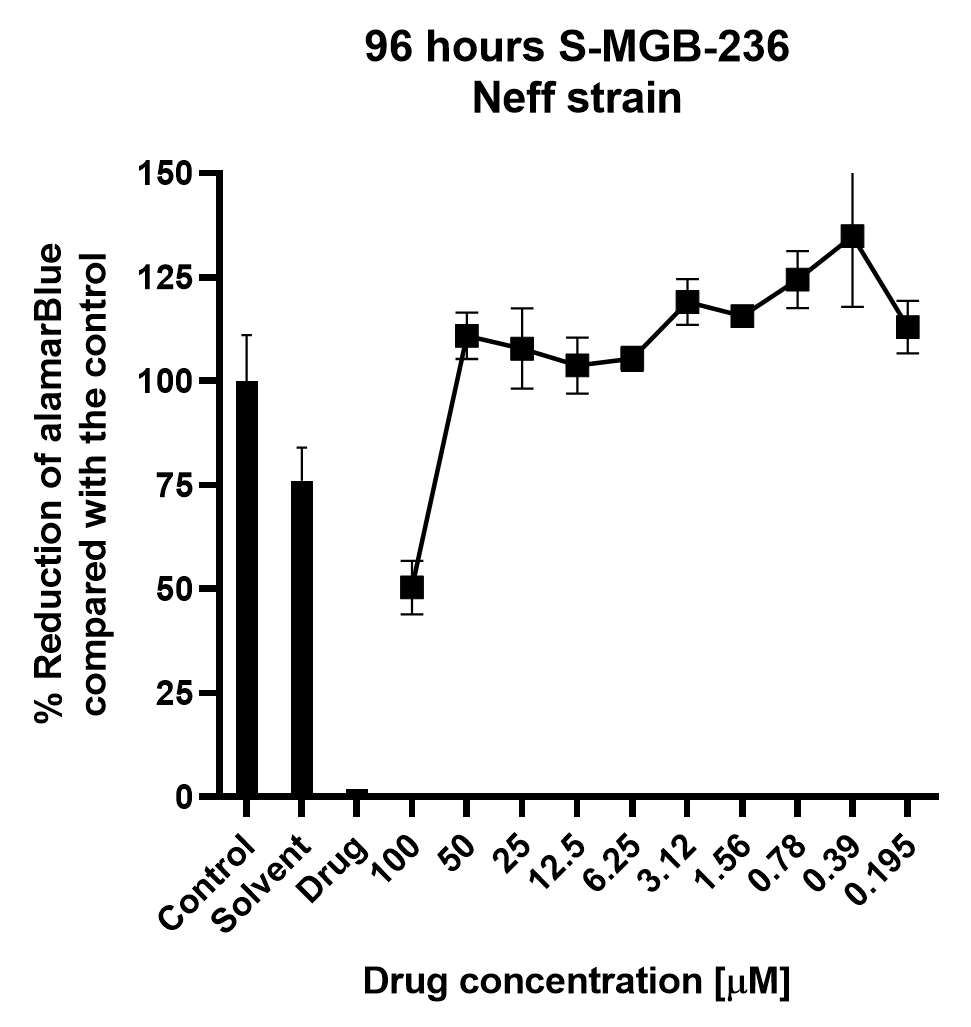


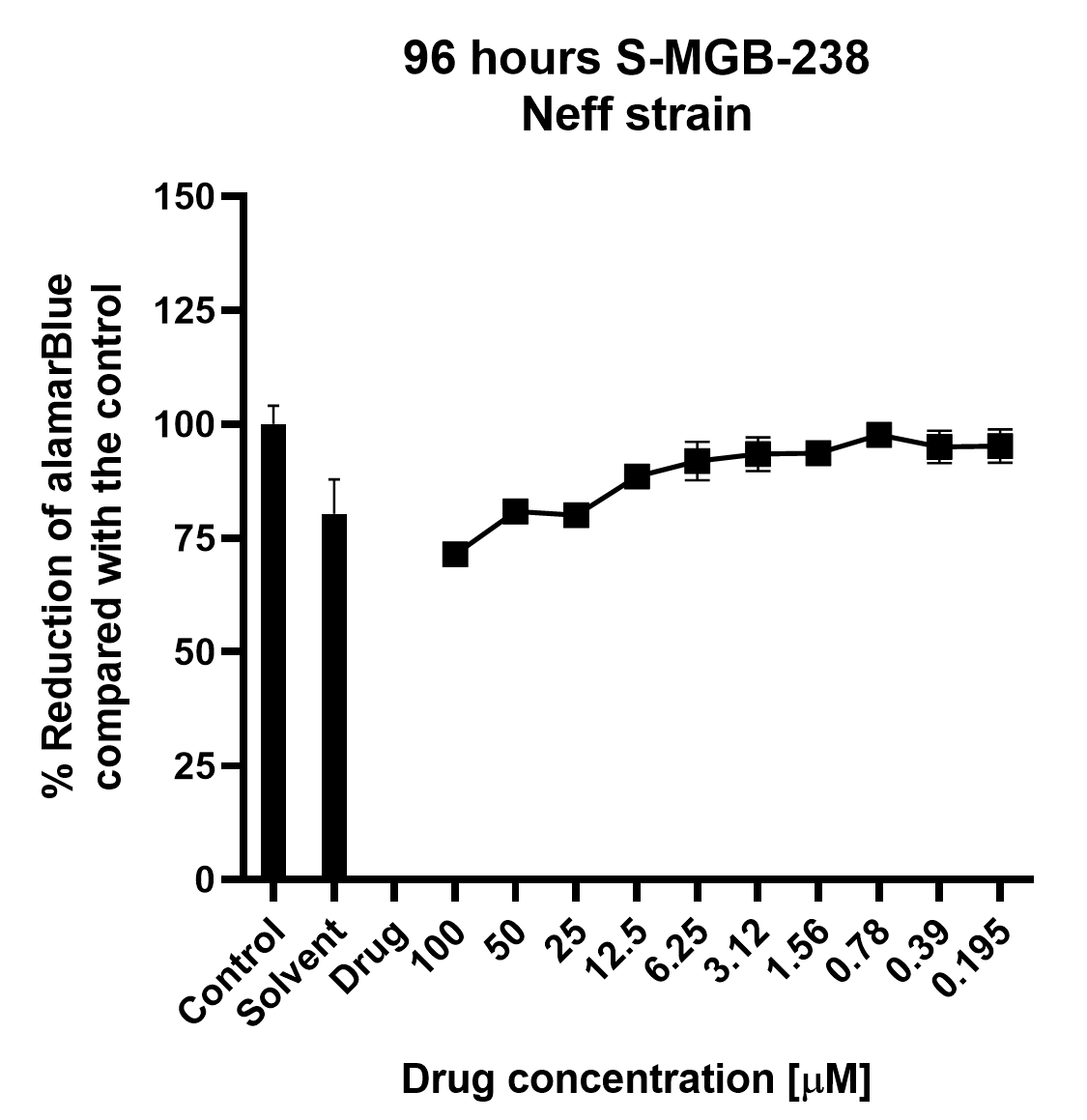

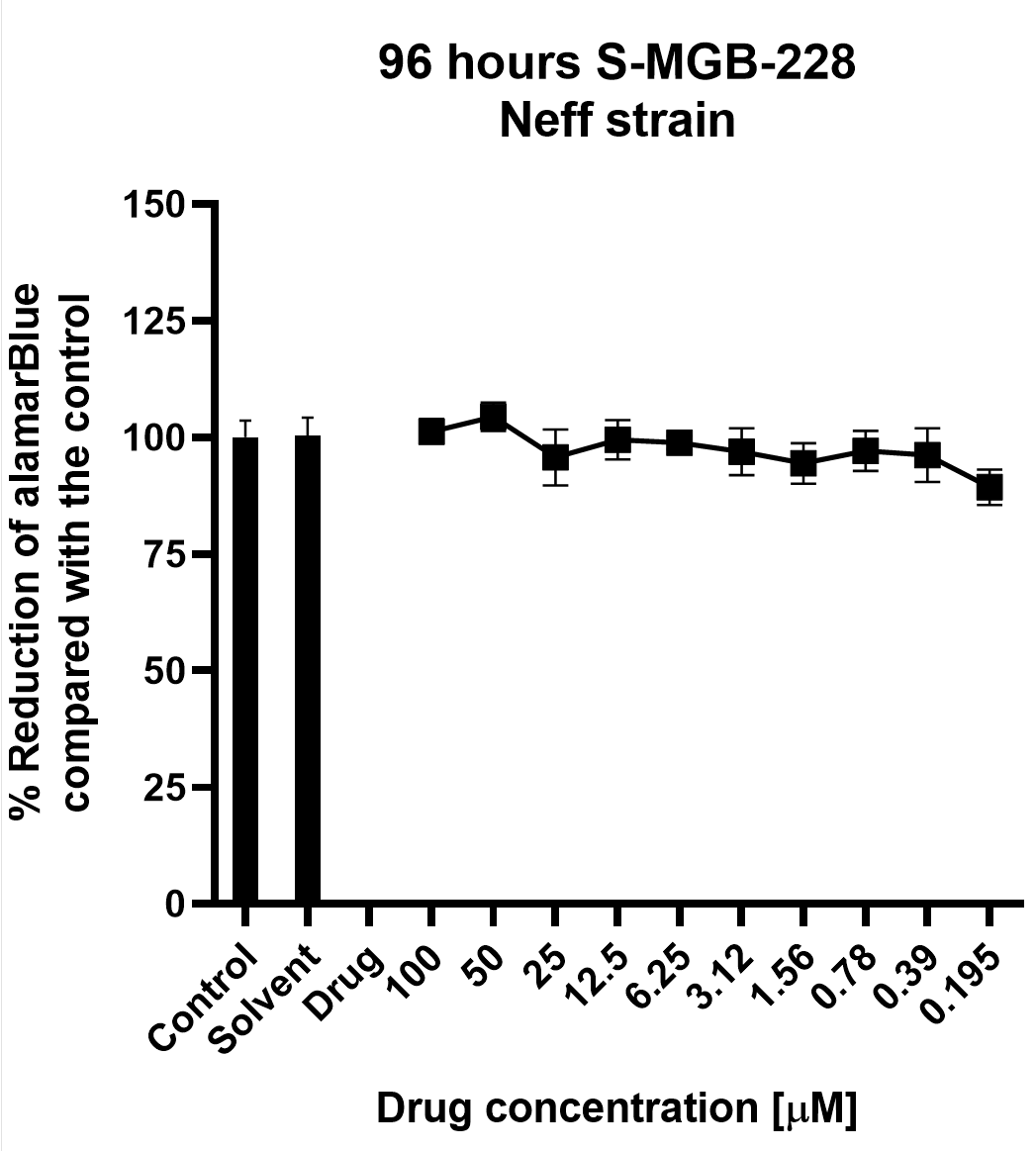

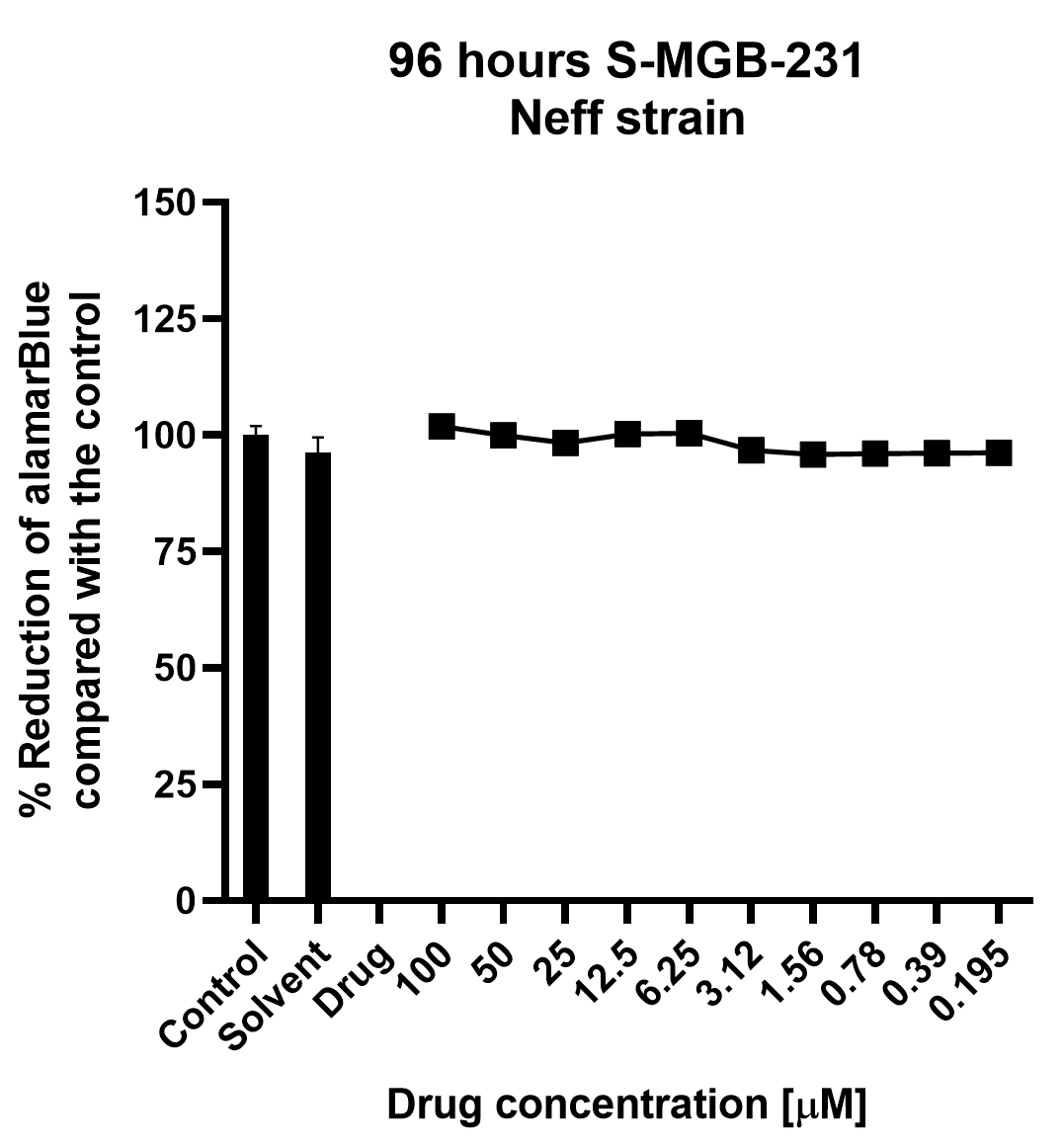


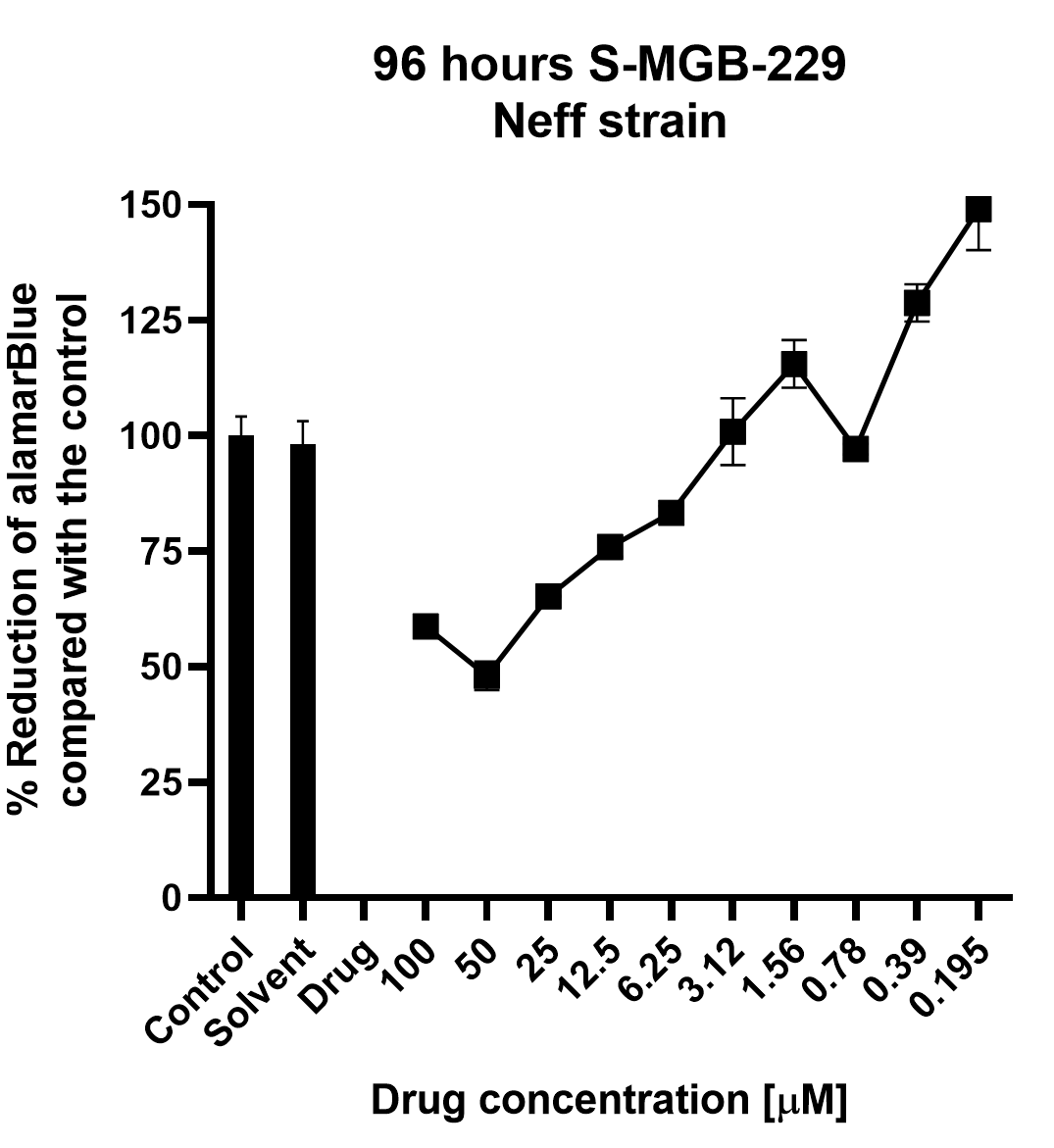

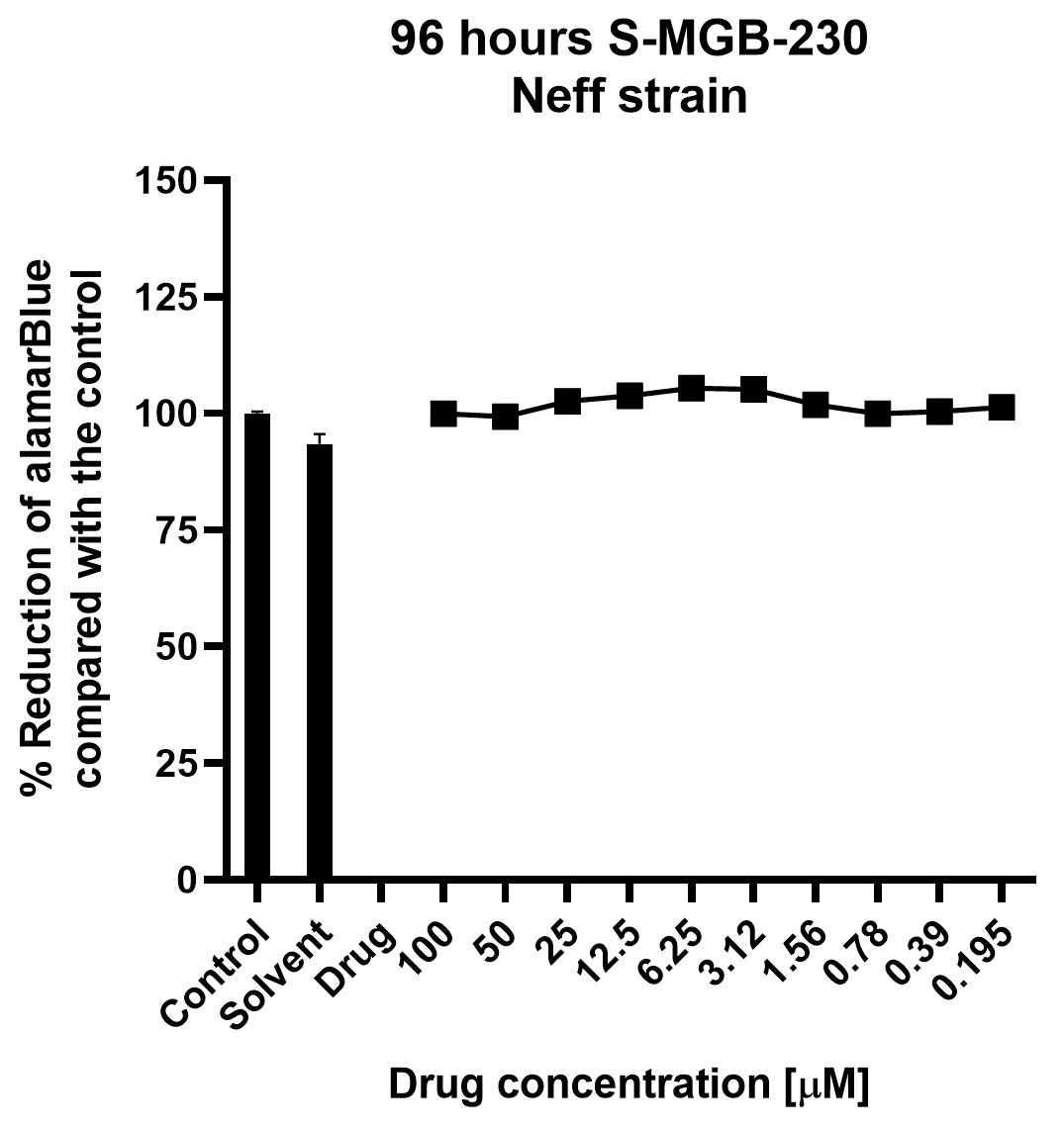

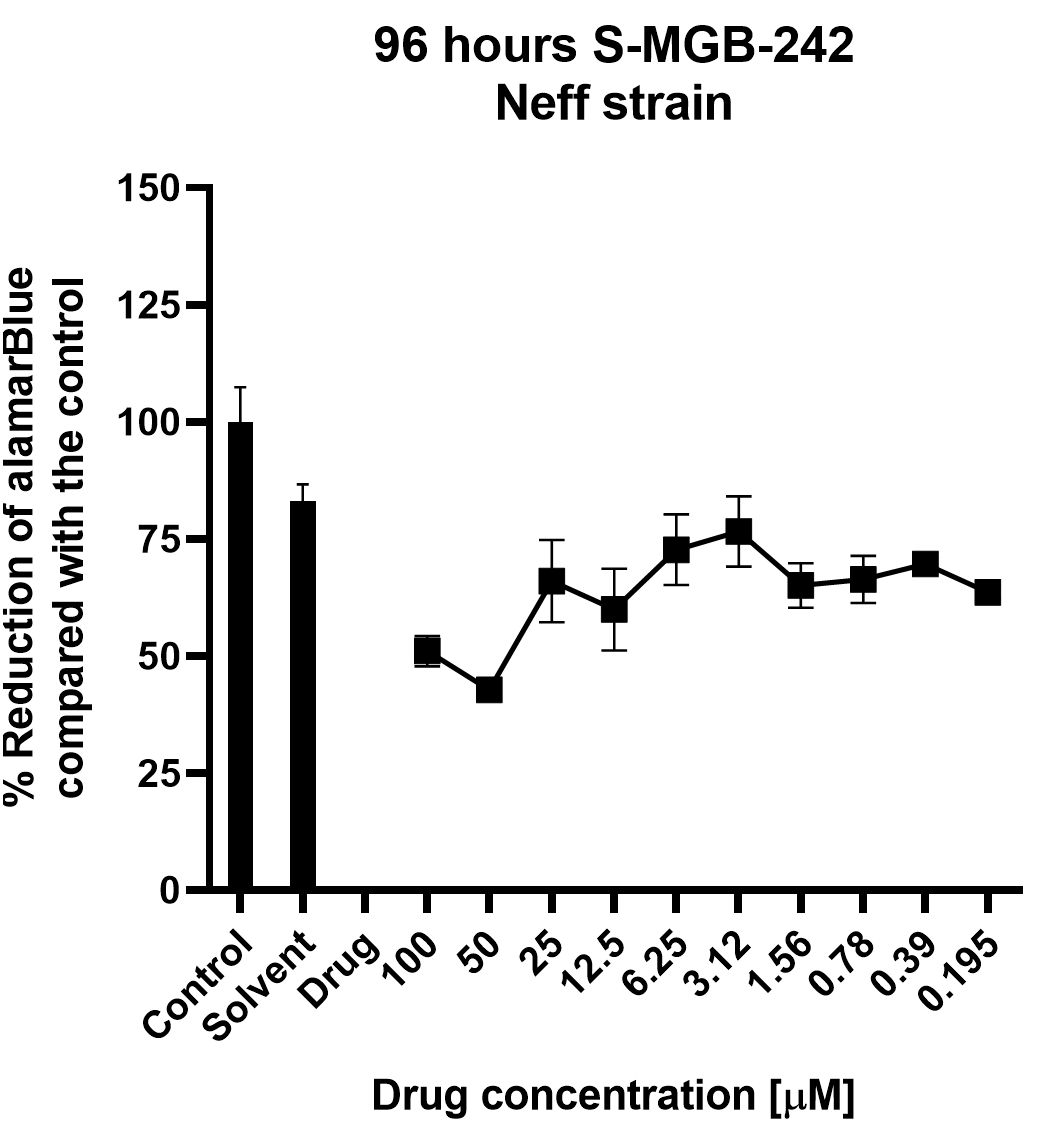


Figure S1: IC_50_ plots of S-MGBs against *A. castellanii* Neff strain through alamarBlue assays. The compounds were tested in triplicate against with a seeding density of 4x10^4^, and incubated at 23°C for 96 hours with alamarBlue™ added at 90 hours. Controls included trophozoites with medium alone (control) and trophozoites with the highest concentration of DMSO (solvent).

**2. Cytotoxicity of S-MGB-241 against HEK293 cells**

Figure S2: HEK293 cytotoxicity was determined at different concentrations (12.5, 25, 50, and 100 µM) of S-MGB-241. The data represented are the mean ±SE percentage of cell survival obtained from three independent experiments in DMEM media containing either galactose or glucose. PrestoBlue™ reagent was used to assess viability of the cells after 24 hours incubation.

**3. 24-hour IC50 Plot of S-MGB-241 against *Acanthamoeba castellanii***

Figure S3: IC_50_ plot of S-MGBs against *A. castellanii* Neff strain through alamarBlue assays. The compounds were tested in triplicate against with a seeding density of 6.4x10^5^cells/ml, and incubated at 23°C for 24 hours with alamarBlue™ added at 90 hours. Controls included trophozoites with medium alone (control) and trophozoites with the highest concentration of DMSO (solvent).

**4. Morphological changes induced by S-MGB-241 at 24 hours treatment**

**
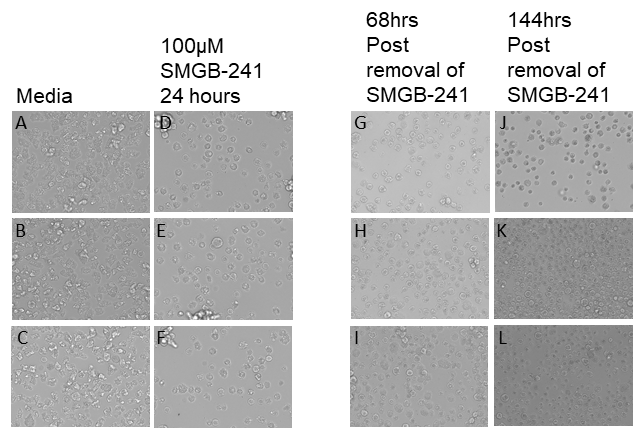
**

Figure S4. Morphological changes induced by S-MGB-241 at 24 hours treatment. Acanthamoeba trophozoites were cultured in media (A-C) or incubated with 100µM S-MGB-241 for 24hrs (D-F). S-MGB-241 was removed and the imaged at 68hrs post removal (G-I) and at 144hrs post removal (J-L)

**5. 96-hour IC_50_ Plot of MGB-BP-3 against *Acanthamoeba castellanii***

Figure S5: IC_50_ plots of S-MGBs against *A. castellanii* Neff strain through alamarBlue assays. The compounds were tested in triplicate against with a seeding density of 2x10^4^/ml, and incubated at 23°C for 96 hours with alamarBlue™ added at 90 hours. Controls included trophozoites with medium alone (control) and trophozoites with the highest concentration of DMSO (solvent).

**6. Synthetic Chemistry**

**6.1 General Methods**

Reagents and Solvents. Used as supplied from Aldrich unless dry DCM or dry THF. These were provided by standard operating procedures for InnovativeTechnology Solvent Purification System.

Solvent Removal. This was carried out by evaporation using a rotary evaporator at reduced pressure (*ca* 20 mmHg) unless otherwise stated.

Thin Layer Chromatography. TLC was carried out using pre-coated silica plates (Alugram^®^ Sil G/UV_254_). Visualisation of TLC plates was achieved by UV (254 nm).

Melting Point. Reichert hot stage melting point apparatus and are uncorrected.

NMR. Bruker Spectrospin 400 MHz or 500 MHz operating frequency for ^1^H NMR nuclei. δ quoted in ppm and measured relative to residual proton from the solvent. Coupling constants, *J,* are given in Hz. _­_^13^C NMR run at 125 MHz and measured relative to the solvent.

IR. Mattson 1000 FTIR spectrometer (Unicam Analytical Systems). Spectrum software. Frequencies are quoted in cm^-1^.

HR-MS-FAB. Recorded on a Jeol JMS-700 M STATION high resolution magnetic sector spectrometer.

Column Chromatography. Silica gel mesh size 230-400 (40-60µm).

Preparative HPLC. Instrument Setup: Waters 1525 Binary HPLC Pump, Waters 717plus Autosampler, Waters 2487 Dual λ Absorbance Detector. LC Conditions: Column, Luna 5µ C18(2) 100A; Dimensions, 60 × 21.1 mm; Injection Volume, 100 µL; Solvent Flow Rate, 6 mL/min; Detection wavelength, 254 nm; solvent A, water (with 0.1% TFA) and solvent B, MeCN (with 0.1% TFA), with gradient changes as per individual compound experimental details.

**6.2 General Procedure for Novel S-MGBs**

Dimer intermediates **18, 19** and **21,** were prepared via previously established methods.^1^ Hydrogenation of appropriate nitro dimer (**18, 19** or **21**) (0.080 mmol, 1.0 Eq) was achieved by Pd/C (10% w/w) in MeOH (3 mL) in the presence of H_2_ for 2 hours. The reaction mixture was subsequently filtered through keisulghur and the solvent removed by rotary evaporation to afford a thin film of residue. This amine intermediate (**22**, **23** or **24**) was taken forward directly without further purification. Amide coupling of the appropriate amino dimer **(22, 23,** or **24)** with the appropriate carboxylic acid **(25** or **26)** (0.080 mmol, 1.0 Eq) in the presence of HBTU (0.17 mmol, 1.5 Eq) in DMF (1 mL) was carried out and left to stir overnight**.** The crude reaction mixture was purified directly by preparative HPLC, followed by freeze drying, to afford the methyl S-MGBs as TFA salts **(27 - 32)** (29 % - 36 %)**.**

The carboxylic acid S-MGBs **(33 - 38)** we obtained by base catalysed hydrolysis of the crude reaction mixture of compounds **(27 - 32)** (0.013 mmol – 0.016 mmol, 1.0 Eq), using lithium hydroxide (0.83 mmol, 59 Eq) in 0.5 mL water for 48 hours**.** These reaction mixtures were purified by preparative HPLC, and fractions containing the desired compounds were subject to freeze drying to afford compounds (**33 – 38)** as a TFA salt (51 %- 68 %). All final S-MGBs were >95% pure as measured by HPLC.

**6.3 Characterisation Data for Novel S-MGBs**

**S-MGB-240, 27, 3-[({4-[({4-[(4-methoxy-4-oxobutanoyl)amino]-1-methyl-1*H*-pyrrol-2-yl}carbonyl)amino]-1-methyl-1*H*-pyrrol-2-yl}carbonyl)amino]-*N*,*N*-dimethyl-1-propanaminium trifluoroacetate.**

*HPLC Procedure*: 0 mins, 90% solvent A: 10% solvent B; 25 mins, 70% solvent A: 30% solvent B; 30 mins, 50% solvent A: 50% solvent B; 35 mins, 90% solvent A: 10% solvent B; Retention time: 15.9 min. *Melting Point:* >230 ^o^C. *IR:* 3279, 2958, 2686, 2563, 1720, 1696, 1681, 1651, 1647, 1638, 1580, 1560, 1206, 1133. *δ_H_ NMR (DMSO-d6):* 9.86 (1H, s), 9.84 (1H, s), 9.21 (1H, bs), 8.14 (1H, t, *J =* 6.0), 7.16 (1H, d, *J =* 1.5), 7.12 (1H, d, *J =* 1.5), 6.93 (1H, bs), 6.88 (1H, d, *J =* 1.5), 3.81 (6H, m), 3.60 (3H, s), 3.24 (2H, m), 3.08 (2H, m), 2.79 (6H, s), 2.54-2.59 (4H, m), 1.84 (2H, m). *HR-MS-FAB:* Found 461.2506 calculated for C_22_H_33_O_5_N_6_^+^ (M+H) 461.2507.

**S-MGB-229, 28, 4-{2-[({4-[({4-[(4-methoxy-4-oxobutanoyl)amino]-1-methyl-1*H*-pyrrol-2-yl}carbonyl)amino]-1-methyl-1*H*-pyrrol-2-yl}carbonyl)amino]ethyl}morpholin-4-ium trifluoroacetate.**

*HPLC Procedure*: 0 mins, 90% solvent A: 10% solvent B; 20 mins, 70% solvent A: 30% solvent B; 25 mins, 50% solvent A: 50% solvent B; 30 mins, 90% solvent A: 10% solvent B; Retention time: 17.5 min. *Melting Point:* >230 ^o^C. *IR:* 3278, 2952, 2879, 1720, 1658, 1645, 1584, 1526, 1468, 1433, 1405, 1373, 1263, 1200, 1174, 1127, 1101. *δ_H_ NMR (DMSO-d6):* 9.86 (1H, s), 9.48 (1H, br), 8.20 (1H, m), 7.17 (1H, d, *J =* 1.5), 7.11 (1H, d, *J =* 1.5), 6.98 (1H, bs), 6.88 (1H, d, *J =* 1.5), 3.98-4.02 (2H, m), 3.86 (6H, m), 3.62-3.68 (2H, m), 3.58 (3H, s), 3.52-3.56 (4H, m), 3.26 (2H, m), 3.12 (2H, m), 2.51-2.54 (4H, m). *HR-MS-FAB:* Found 489.2450 calculated for C_23_H_33_O_6_N_6_^+^ (M+H) 489.2456.

**S-MGB-231, 29, 1-amino-3-[({4-[({4-[(4-methoxy-4-oxobutanoyl)amino]-1-methyl-1*H*-pyrrol-2-yl}carbonyl)amino]-1-methyl-1*H*-pyrrol-2-yl}carbonyl)amino]-1-propaniminium trifluoroacetate**

*HPLC Procedure*: 0 mins, 90% solvent A: 10% solvent B; 25 mins, 70% solvent A: 30% solvent B; 30 mins, 50% solvent A: 50% solvent B; 35 mins, 90% solvent A: 10% solvent B; Retention time: 15.1 min. *Melting Point*: >230 ^o^C. *IR*: 3303, 3116, 2962, 1740, 1725, 1701, 1686, 1660, 1643, 1632, 1580, 1543, 1528, 1470, 1435, 1412, 1367, 1259, 1198, 1140. *δ_H_ NMR (DMSO-d6):* 9.89 (1H, s), 9.84 (1H, s), 8.88 (2H, s), 8.42 (2H, s), 8.17 (1H, t, *J =* 6.0), 7.14 (1H, d, *J =* 1.5), 7.11 (1H, d, *J =* 1.5), 6.93 (1H, bs), 6.87 (1H, d, *J =* 1.5), 3.81 (6H, m), 3.58 (3H, s), 3.48 (2H, m), 2.57-2.60 (2H, m), 2.51-2.54 (4H, m). *HR-MS-FAB:* Found 446.2150 calculated for C_20_H_28_O_5_N_7_^+^ (M+H) 446.2146.

**S-MGB-238, 30, 3-{[(4-{[(4-{[4-(methoxycarbonyl)benzoyl]amino}-1-methyl-1*H*-pyrrol-2-yl)carbonyl]amino}-1-methyl-1*H*-pyrrol-2-yl)carbonyl]amino}-*N*,*N*-dimethyl-1-propanaminium trifluoroacetate.**

*HPLC Procedure*: 0 mins, 90% solvent A: 10% solvent B; 25 mins, 70% solvent A: 30% solvent B; 30 mins, 50% solvent A: 50% solvent B; 35 mins, 90% solvent A: 10% solvent B; Retention time: 22.1 min. *Melting Point:* >230 ^o^C. *IR:* 3323, 2945, 2880, 2658, 2499, 1701, 1683, 1668, 1629, 1619, 1558, 1496, 1196, 1135. *δ_H_ NMR (DMSO-d6):* 10.53 (1H, s), 9.96 (1H, s), 9.18 (1H, m), 8.15 (1H, m), 8.04-8.10 (4H, m), 7.34 (1H, d, *J =* 1.5), 7.18 (1H, d, *J =* 1.5), 7.12 (1H, d, *J =* 1.5), 6.96 (1H, d, *J =* 1.5), 3.91 (3H, s), 3.89 (3H, s), 3.83 (3H, s), 3.24 (2H, m), 3.08 (2H, m), 2.80 (6H, s), 1.85 (2H, m). *HR-MS-FAB:* Found 509.2511 calculated for C_26_H_33_O_5_N_6_^+^ (M+H) 509.2507.

**S-MGB-227, 31, 4-(2-{[(4-{[(4-{[4-(methoxycarbonyl)benzoyl]amino}-1-methyl-1*H*-pyrrol-2-yl)carbonyl]amino}-1-methyl-1*H*-pyrrol-2-yl)carbonyl]amino}ethyl)morpholin-4-ium trifluoroacetate.**

*HPLC Procedure*: 0 mins, 90% solvent A: 10% solvent B; 20 mins, 70% solvent A: 30% solvent B; 25 mins, 50% solvent A: 50% solvent B; 30 mins, 90% solvent A: 10% solvent B; Retention time: 22.0 min. *Melting Point:* >230 ^o^C. *IR:* 3299, 2965, 1675, 1645, 1623, 1580, 1533, 1468, 1433, 1407, 1280, 1198, 1183, 1129, 1092, 1015, 1011. *δ_H_ NMR (DMSO-d6):* 10.53 (1H, s), 9.98 (1H, s), 9.50 (1H, bs), 8.22 (1H, m), 8.04-8.10 (4H, m), 7.33 (1H, s), 7.20 (1H, s), 7.11 (1H, s), 7.00 (1H, s), 3.98-4.02 (2H, m), 3.89 (3H, s), 3.87 (3H, s), 3.83 (3H, s), 3.62-3.68 (2H, m), 3.52-3.56 (4H, m), 3.24 (2H, m), 3.13 (2H, m). *HR-MS-FAB:* Found 537.2450 calculated for C_27_H_33_O_6_N_6_^+^ (M+H) 537.2456.

**S-MGB-233, 32, Synthesis of 1-amino-3-{[(4-{[(4-{[4-(methoxycarbonyl)benzoyl]amino}-1-methyl-1*H*-pyrrol-2-yl)carbonyl]amino}-1-methyl-1*H*-pyrrol-2-yl)carbonyl]amino}-1-propaniminium trifluoroacetate.**

*HPLC Procedure*: 0 mins, 90% solvent A: 10% solvent B; 25 mins, 80% solvent A: 20% solvent B; 30 mins, 50% solvent A: 50% solvent B; 35 mins, 90% solvent A: 10% solvent B; Retention time: 20.8 min. *Melting Point:* >230 ^o^C. *IR:* 3299, 3103, 2943, 1707, 1688, 1675, 1660, 1645, 1634, 1623, 1580, 1565, 1539, 1530, 1507, 1494, 1440, 1435, 1407, 1394, 1280, 1187, 1121, 1058, 1015. *δ_H_ NMR (DMSO-d6):* 10.53 (1H, s), 9.95 (1H, s), 8.89 (2H, s), 8.43 (2H, s), 8.18 (1H, m), 8.04-8.10 (4H, m), 7.33 (1H, d, *J =* 1.5), 7.17 (1H, d, *J =* 1.5), 7.11 (1H, d, *J =* 1.5), 6.95 (1H, d, *J =* 1.5), 3.89 (3H, s), 3.87 (3H, s), 3.81 (3H, s), 3.46-3.52 (2H, m), 2.58-2.61 (2H, m). *HR-MS-FAB:* Found 494.2148 calculated for C_24_H_28_O_5_N_7_^+^ (M+H) 494.2146

**S-MGB-242, 33, 3-[({4-[({4-[(3-carboxypropanoyl)amino]-1-methyl-1*H*-pyrrol-2-yl}carbonyl)amino]-1-methyl-1*H*-pyrrol-2-yl}carbonyl)amino]-*N*,*N*-dimethyl-1-propanaminium trifluoroacetate.**

*HPLC Procedure*: 0 mins, 90% solvent A: 10% solvent B; 25 mins, 70% solvent A: 30% solvent B; 30 mins, 50% solvent A: 50% solvent B; 35 mins, 90% solvent A: 10% solvent B; Retention time: 15.1 min. *Melting Point: >*230 ^o^C. *IR:* 3256, 2956, 2660, 2566, 1720, 1698, 1685, 1647, 1636, 1575, 1534, 1414, 1282, 1196, 1109. *δ_H_ NMR (DMSO-d6):* 12.08 (1H, s), 9.83 (2H, bs), 9.21 (1H, bs), 8.14 (1H, t, *J =* 6.0), 7.16 (1H, d, *J =* 1.5), 7.12 (1H, d, *J =* 1.5), 6.93 (1H, bs), 6.88 (1H, d, *J =* 1.5), 3.83 (6H, m), 3.24 (2H, m), 3.07 (2H, m), 2.79 (6H, s), 2.54-2.59 (4H, m), 1.84 (2H, m). *HR-MS-FAB:* Found 447.2351 calculated for C_21_H_31_O_5_N_6_^+^ (M+H) 447.2350.

**S-MGB-230, 34, 4-{2-[({4-[({4-[(3-carboxypropanoyl)amino]-1-methyl-1*H*-pyrrol-2-yl}carbonyl)amino]-1-methyl-1*H*-pyrrol-2-yl}carbonyl)amino]ethyl}morpholin-4-ium trifluoroacetate.**

*HPLC Procedure*: 0 mins, 90% solvent A: 10% solvent B; 20 mins, 70% solvent A: 30% solvent B; 25 mins, 50% solvent A: 50% solvent B; 30 mins, 90% solvent A: 10% solvent B; Retention time: 16.1 min. *Melting Point:* >230 ^o^C. *IR:* 3265, 3129, 2939, 2613, 2497, 1700, 1688, 1657, 1645, 1638, 1627, 1582, 1539, 1511, 1127. *δ_H_ NMR (DMSO-d6):* 12.08 (1H, bs), 9.87 (1H, s), 9.83 (1H, s), 9.56 (1H, bs), 8.20 (1H, m), 7.17 (1H, s), 7.11 (1H, d, *J =* 1.5), 6.98 (1H, s), 6.88 (1H, s), 3.98-4.02 (2H, m), 3.81 (3H, m), 3.62-3.68 (2H, m), 3.583 (3H, s), 3.52-3.56 (4H, m), 3.24 (2H, m), 3.13 (2H, m), 2.51-2.54 (4H, m). *HR-MS-FAB:* Found 475.2300 calculated for C_22_H_31_O_6_N_6_^+^ (M+H) 475.2300.

**S-MGB-236, 35,** **1-amino-3-[({4-[({4-[(3-carboxypropanoyl)amino]-1-methyl-1*H*-pyrrol-2-yl}carbonyl)amino]-1-methyl-1*H*-pyrrol-2-yl}carbonyl)amino]-1-propaniminium trifluoroacetate.**

*HPLC Procedure*: 0 mins, 90% solvent A: 10% solvent B; 25 mins, 70% solvent A: 30% solvent B; 30 mins, 50% solvent A: 50% solvent B; 35 mins, 90% solvent A: 10% solvent B; Retention time: 14.5 min. *Melting Point*: >230 ^o^C. *IR:* 3303, 3064, 2945, 1740, 1705, 1686, 1677, 1662, 1656, 1645, 1634, 1625, 1582, 1573, 1556, 1553, 1405, 1276, 1202, 1185. *δ_H_ NMR (DMSO-d6):* 12.07 (1H, bs), 9.83-9.86 (2H, m), 8.89 (2H, s), 8.44 (2H, s), 8.18 (1H, t, *J =* 6.0), 7.16 (1H, d, *J =* 1.5), 7.12 (1H, d, *J =* 1.5), 6.95 (1H, d, *J =* 1.5), 6.89 (1H, d, *J =* 1.5), 3.83 (6H, m), 3.49 (2H, m), 2.57-2.60 (2H, m), 2.51-2.54 (4H, m). *HR-MS-FAB:* Found 432.1992 calculated for C_19_H_26_O_5_N_7_^+^ (M+H) 432.1990.

**S-MGB-241, 36, Synthesis of 3-[({4-[({4-[(4-carboxybenzoyl)amino]-1-methyl-1*H*-pyrrol-2-yl}carbonyl)amino]-1-methyl-1*H*-pyrrol-2-yl}carbonyl)amino]-*N*,*N*-dimethyl-1-propanaminium trifluoroacetate.**

*HPLC Procedure*: 0 mins, 90% solvent A: 10% solvent B; 20 mins, 70% solvent A: 30% solvent B; 25 mins, 50% solvent A: 50% solvent B; 30 mins, 90% solvent A: 10% solvent B; Retention time: 20.7 min. *Melting Point:* >230 ^o^C. *IR:* 3327, 2956, 2660, 2553, 1733, 1701, 1687, 1575, 1541, 1468, 1407, 1206, 1114. *δ_H_ NMR (DMSO-d6):* 10.50 (1H, s), 9.95 (1H, s), 9.18 (1H, m), 8.15 (1H, m), 8.03-8.09 (4H, m), 7.34 (1H, d, *J =* 1.5), 7.19 (1H, d, *J =* 1.5), 7.12 (1H, d, *J =* 1.5), 6.95 (1H, d, *J =* 1.5), 3.89 (3H, s), 3.83 (3H, s), 3.25 (2H, m), 3.08 (2H, m), 2.79 (6H, s), 1.85 (2H, m). *δ_C_* *NMR (126 Hz, DMSO-d_6_)*: 167.22, 163.53, 162.13, 158.95, 138.88, 133.45, 129.84, 128.12, 123.56, 123.02, 122.69, 122.42, 119.41, 118.62, 105.33, 105.02, 55.31, 42.81, 36.66, 36.53, 35.95, 25.24. *HR-MS-FAB:* Found 495.2350 calculated for C_25_H_31_O_5_N_6_^+^ (M+H) 495.2324.

**S-MGB-228, 37, Synthesis of 4-{2-[({4-[({4-[(4-carboxybenzoyl)amino]-1-methyl-1*H*-pyrrol-2-yl}carbonyl)amino]-1-methyl-1*H*-pyrrol-2-yl}carbonyl)amino]ethyl}morpholin-4-ium trifluoroacetate.**

*HPLC Procedure*: 0 mins, 90% solvent A: 10% solvent B; 20 mins, 70% solvent A: 30% solvent B; 25 mins, 50% solvent A: 50% solvent B; 30 mins, 90% solvent A: 10% solvent B; Retention time: 20.6 min. *Melting Point:* >230 ^o^C. *IR:* 3223, 2945, 2889, 1675, 1643, 1584, 1530, 1464, 1438, 1410, 1263, 1177, 1129, 1092, 1052, 1009. *δ_H_ NMR (DMSO-d6):* 13.21 (1H, s), 10.49 (1H, s), 9.98 (1H, s), 9.49 (1H, bs), 8.22 (1H, m), 8.02-8.07 (4H, m), 7.33 (1H, s), 7.20 (1H, s), 7.12 (1H, s), 7.00 (1H, s), 3.99-4.03 (2H, m), 3.88 (3H, s), 3.83 (3H, s), 3.62-3.68 (2H, m), 3.52-3.56 (4H, m), 3.23 (2H, m), 3.12 (2H, m). *HR-MS-FAB:* Found 523.2294 calculated for C_26_H_31_O_6_N_6_^+^ (M+H) 523.2300.

**S-MGB-237, 38, 1-amino-3-[({4-[({4-[(4-carboxybenzoyl)amino]-1-methyl-1*H*-pyrrol-2-yl}carbonyl)amino]-1-methyl-1*H*-pyrrol-2-yl}carbonyl)amino]-1-propaniminium trifluoroacetate.**

*HPLC Procedure*: 0 mins, 90% solvent A: 10% solvent B; 25 mins, 80% solvent A: 20% solvent B; 30 mins, 50% solvent A: 50% solvent B; 35 mins, 90% solvent A: 10% solvent B; Retention time: 18.2 min. *Melting Point:* >230 ^o^C. *IR:* 3305, 3193, 2939, 1703, 1668, 1653, 1634, 1576, 1545, 1515, 1477, 1464, 1435, 1405, 1289, 1261, 1203, 1131, 1114, 1062, 1023. *δ_H_ NMR (DMSO-d6):* 13.20 (1H, bs), 10.50 (1H, s), 9.94 (1H, s), 8.03-8.09 (4H, m), 7.98 (1H, m), 7.35 (1H, s, *J =* 1.5), 7.33 (2H, m), 7.21 (1H, d, *J =* 1.5), 7.10 (1H, ds, *J =* 1.5), 6.84 (1H, d, *J =* 1.5), 6.81 (2H, m), 3.88 (3H, s), 3.81 (3H, s), 3.34-3.38 (2H, m), 2.31-2.35 (2H, m). *HR-MS-FAB:* Found 480.1995 calculated for C_23_H_26_O_5_N_7_^+^ (M+H) 480.1990.

**6.4 Biophysical Studies of S-MGB-241**

*Native Mass Spectrometry*

| **Species** | **m/z value** | **Calculated mass of neutral species (Da)** |
| --- | --- | --- |
| **Single Stranded [SS]** | 3- : 1214.1  4- : 910.3 | (1214.1*3) + 3 = 3645.3  (910.3*4) + 4 = 3645.2 |
| **Double Stranded [DS]** | 4- : 1821.7  5- : 1457.2 | (1821.7*4) + 4 = 7290.8  (1457.2*5) + 5 = 7291.0 |
| ***Double Stranded + 2 x S-MGB-241 [DS+2M]*** | 4- : 2069.0  5- : 1655.0 | (2069.0*4) + 4 = 8280.0  (1655.0*5) +5 = 8280.0 |

**Table S1:** Calculated and measured masses for each species observed in **Figure 2** for DNA sequence 5’-CGCATATATGCG-3’ **S-MGB-241, 36.**

| **S-MGB-241** | **Model** | **Equation** | **A1** | **A2** | **X_0_** | **dx** | **Reduced Chi-Sqr** | **R-Square (COD)** | **Adj. R-Square** |
| --- | --- | --- | --- | --- | --- | --- | --- | --- | --- |
| **gDNA 1** | Boltzmann | y = A2 + (A1-A2)/(1 + exp((x-x0)/dx)) | 0.01157 ± 0.00499 | 0.93859 ± 0.0056 | 71.4973 ± 0.11862 | 3.46957 ± 0.10614 | 6.80379E-4 | 0.99581 | 0.99568 |
| **COM**  **1** | Boltzmann | y = A2 + (A1-A2)/(1 + exp((x-x0)/dx)) | 0.12587 ± 0.00313 | 0.98479 ± 0.00346 | 71.77417 ± 0.06527 | 2.08488 ± 0.0568 | 3.77101E-4 | 0.99764 | 0.99757 |
| **gDNA 2** | Boltzmann | y = A2 + (A1-A2)/(1 + exp((x-x0)/dx)) | -0.04914 ± 0.00207 | 0.98553 ± 0.00215 | 70.66127 ± 0.0352 | 2.14493 ± 0.03065 | 1.53576E-4 | 0.99934 | 0.99932 |
| **COM**  **2** | Boltzmann | y = A2 + (A1-A2)/(1 + exp((x-x0)/dx)) | 0.06902 ± 0.00284 | 1.02275 ± 0.00312 | 71.69482 ± 0.05015 | 1.8293 ± 0.04359 | 3.25692E-4 | 0.99839 | 0.99834 |
| **gDNA**  **3** | Boltzmann | y = A2 + (A1-A2)/(1 + exp((x-x0)/dx)) | -0.01719 ± 0.00221 | 0.97986 ± 0.00231 | 70.7207 ± 0.03842 | 2.0567 ± 0.03343 | 1.7959E-4 | 0.99917 | 0.99915 |
| **COM**  **3** | Boltzmann | y = A2 + (A1-A2)/(1 + exp((x-x0)/dx)) | 0.06681 ± 0.00218 | 0.96316 ± 0.00246 | 72.04076 ± 0.0465 | 2.36814 ± 0.04057 | 1.75011E-4 | 0.99896 | 0.99893 |
| **gDNA 4** | Boltzmann | y = A2 + (A1-A2)/(1 + exp((x-x0)/dx)) | -0.01607 ± 0.00186 | 0.97225 ± 0.00199 | 71.09383 ± 0.03432 | 2.25445 ± 0.02991 | 1.24423E-4 | 0.9994 | 0.99938 |
| **COM**  **4** | Boltzmann | y = A2 + (A1-A2)/(1 + exp((x-x0)/dx)) | 0.04701 ± 0.00188 | 0.9716 ± 0.00207 | 71.6063 ± 0.03752 | 2.23463 ± 0.03269 | 1.31537E-4 | 0.99928 | 0.99926 |

**Table S2:** gDNA and **gDNA:S-MGB-241** complex graph model details. gDNA: DNA with no S-MGB present. COM: gDNA + S-MGB present.

*DNA Thermal Shift*

Melting temperatures of gDNA and **gDNA:S-MGB-241** complex calculated from fitted Boltzmann distributions using OriginPro 2021. All values are an average for n = 4 experimental repeats.

| **RUN ID** | **gDNA X_0_** | **gDNA:S-MGB-241 Complex X_0_** | **Difference (^o^C)** |
| --- | --- | --- | --- |
| **1** | 71.50 | 71.77 | 0.27 |
| **2** | 70.66 | 71.69 | 1.03 |
| **3** | 70.72 | 72.04 | 1.32 |
| **4** | 71.09 | 71.61 | 0.52 |

**Table S3: S-MGB-241** complex experiment results **X_0_** values.

|  | **gDNA X_0_** | **gDNA:S-MGB-241 Complex X_0_** |
| --- | --- | --- |
| **1** | 71.5 | 71.69 |
| **2** | 70.66 | 71.77 |
| **3** | 70.72 | 71.61 |
| **4** | 71.09 | 72.04 |
| **Average** | 70.9925 | 71.775 |
| **Standard Deviation** | 0.388104 | 0.189824 |
| **Standard Error** | 0.194052 | 0.094912 |

**Table S4:** Mathematical values used to determine statistical difference between **dDNA X_0_** and **gDNA: S-MGB-241 complex X_0_.**

*T-test:* The *t*-value is -3.62235. The *p*-value is 0.011066. The result is significant at *p* < 0.05.

**7. References**

1. Scott, F.J., Khalaf, A.I., Giordani, F., Wong, P.E., Duffy, S., Barrett, M., Avery, V.M., Suckling, C.J., 2016. An evaluation of Minor Groove Binders as anti- Trypanosoma brucei brucei therapeutics. European Journal of Medicinal Chemistry 116, 116–125.. https://doi.org/10.1016/j.ejmech.2016.03.064
